# Disclosure experiences in LGBTQ+ healthcare staff: a systematic review and meta-synthesis

**DOI:** 10.1136/bmjopen-2025-100412

**Published:** 2026-03-04

**Authors:** Reem Prakkash, Lucy Manning, Laia Bécares, Stephani L Hatch, Isaac Akande, Sarah Dorrington

**Affiliations:** 1 Department of Psychosis Studies, King’s College London, London, UK; 2 Department of Psychological Medicine, King’s College London, London, UK; 3 South London and Maudsley NHS Foundation Trust, London, UK; 4 Department of Global Health & Social Medicine, King’s College London, London, UK; 5 ESRC Centre for Society and Mental Health, King’s College London, London, UK

**Keywords:** Organisational development, Health Workforce, MENTAL HEALTH, Occupational Stress, Sexual and Gender Minorities, Systematic Review

## Abstract

**Abstract:**

**Objective:**

Workplace disclosure of Lesbian, Gay, Bisexual, Transgender and Queer (LGBTQ+) identity by healthcare employees is an understudied area and existing reviews of LGBTQ+ disclosure in the healthcare sector focus on patient perspectives, overlooking the unique challenges that healthcare professionals encounter. The aim of this study was to conduct a systematic review and meta-synthesis of existing qualitative studies exploring disclosure experiences of LGBTQ+ healthcare employees.

**Method:**

The literature search integrated current research from 2011 to March 2023 and focused on qualitative studies exploring disclosure experiences of LGBTQ+ healthcare professionals. Ovid served as the primary platform for literature searches, supplemented by forward and backward citation tracking and additional searches in academic databases such as Google Scholar and Scopus. The studies underwent quality evaluation using the Critical Appraisal Skills Programme 2022 checklist and were synthesised using thematic analysis.

**Results:**

The findings revealed seven studies with five prominent themes: (1) risk associated with disclosure, (2) making the decision to disclose, (3) cost of non-disclosure, (4) cost of disclosure and (5) benefit of disclosure. Additionally, five critical factors of disclosure were identified: level, scope, time, elements and method. Finally, the risk–benefit analysis underscored the dilemma and balance between authenticity and conformity, largely influenced by pervasive heteronormativity, resulting in a significant mental toll.

**Conclusions:**

The findings must be interpreted considering certain limitations, such as the lack of generalisability of studies. However, the findings emphasise the critical need for cultivating trusting and accepting healthcare work environments for LGBTQ+ staff.

**STRENGTHS AND LIMITATIONS OF THIS STUDY**

- Two independent coders conducted thematic synthesis, coding separately and then comparing findings to ensure consistency, focusing on explicit contexts to reduce bias.
- Researchers have openly reflected on their positionality when analysing themes.
- The study includes only research from countries that endorsed the Lesbian, Gay, Bisexual and Transgender rights declaration in the Human Rights Council in 2011; studies are from 2011 to 2023.
- Meta-synthesis allowed for a deep exploration of themes and nuances, providing insights into the workplace disclosure experiences of Lesbian, Gay, Bisexual, Transgender and Queer healthcare employees.
- Thorough literature search of major electronic databases and studies underwent quality evaluation using the Critical Appraisal Skills Programme 2022 checklist.

## Introduction

 Exposure to discrimination is common among Lesbian, Gay, Bisexual, Transgender and Queer (LGBTQ+) healthcare professionals and is associated with distress and burnout in a workforce already under strain due to long working hours and high demand.^1 2^ Empirical studies consistently show that a significant number of LGBTQ+ (lesbian, gay, bi, trans, queer, questioning, intersex, asexual and more; henceforth referred to as LGBTQ+) employees encounter additional workplace challenges based on their sexual orientation and gender identity, with 44% experiencing discrimination and 13% reporting verbal harassment in their work environments.^3^,^6^ LGBTQ+ healthcare professionals encounter unique workplace challenges linked to bias and how to navigate their sexual orientation and/or gender identities within the workplace. Consequently, these attributes make LGBTQ+ healthcare staff particularly susceptible to developing poor physical and mental health.^7 8^

While there is limited research examining health outcomes among LGBTQ+ healthcare staff, it has been well-established that LGBTQ+ employees often experience poorer well-being in various other workplace settings.^8^,^10^ LGBTQ+ healthcare workers may experience discomfort in revealing their identity and frequently find themselves in a dilemma regarding disclosure at work.^8 11^ In healthcare workplaces, the systems of oppression remain a reality, and concealing their LGBTQ+ identities can be a way for healthcare workers to safeguard their professional status and emotional well-being.^12^ This has created a context whereby LGBTQ+ professionals working in the healthcare industry may opt for non-disclosure in the workplace, which can protect against stigma connected with sexual orientation and/or gender identity.^713^,^15^ However, concealment can also be associated with feelings of alienation, distress and shame.^16^

A study by Eliason *et al*, drawing on respondents from US professional networks including GLMA: Health Professionals Advancing LGBT Equality, identified that 38% of sexual and gender minority healthcare staff conceal their identity in the workplace,^7^ highlighting an urgent need to reassess the present workplace conditions. Concerns about potential loss of employment and career prospects, violence and discrimination, along with discomfort brought on by cis-heteronormative attitudes, have been the main influencers behind this decision.^7 17^ Cis-heteronormativity is a pervasive belief system that asserts heterosexuality and cisgender identity as the standard mode of existence.^18^ As a result, this presumption can lead to a marginalisation and disregard of non-heterosexual identities. In an environment dominated by heteronormativity, research indicates that LGBTQ+ employees are frequently compelled to assess risks carefully and withhold full disclosure about their identities to avoid discomfort or potential negative consequences.^13 19^ While assessing the potential risks and navigating their personal and professional identities at work, LGBTQ+ workers often face significant conflicts related to disclosure, particularly when confronted with non-accepting circumstances. In such situations, they may find it challenging to disclose their identity, leading them to operate and juggle between two distinct identities simultaneously.^20^

### Understanding disclosure

#### Definition and conceptualisation

The literature on disclosure applies both the terms ‘disclosure’ and ‘coming out’. While disclosure is often defined as being more process and space driven, coming out has been defined as acknowledging and communicating a marginalised non-heterosexual and non-cisgender identity to others and oneself.^621^,^23^ In keeping with the above, and the use of both terms within the LGBTQ+ community, this paper will use the terms ‘disclosure’ and ‘coming out’ interchangeably.

Early definitions of disclosure describe it as the act of expressing one’s sensitive information.^24^,^26^ However, current scholars believe that prior literature overlooked and simplified the process, thereby disregarding the significant role it plays in an individual’s life.^23^ Gray^27^ describes coming out as a continuous process, rather than a one-time experience. Similarly, the understanding of disclosure has considerably expanded and now encompasses the continuous awareness of self-identity, the process of determining and sharing the intricate information, while recognising one’s role within the larger social framework.^28 29^ More specifically, within the workplace, individuals may make a deliberate choice regarding the disclosure of their sexual orientation and/or gender identity as a way of controlling and managing their visibility.^20^

#### Background and literature

Social identity theory argues that individuals classify themselves into distinct groups, influencing their self-perception and thought processes.^30^,^32^ Diversity in the workplace can evoke positive emotions among in-group members but have the opposite impact on the out-group,^14 30^ potentially influencing disclosure decisions. Consequently, when faced with discrimination from co-workers, it can become challenging for LGBTQ+ individuals to satisfy their basic needs of belongingness and recognition,^33^,^35^ which have been established as fundamental for disclosure.^36^,^38^ Research on systemic injustice further sheds light on the harmful effects stemming from exclusion, marginalisation and minority status.^39 40^

As a result, professionals adopt different ways of navigating disclosure to manage their identities in the workplace. Within professional environments, individuals often disclose their stigmatised identities on a continuum, involving various degrees of concealing or expressing their identity. Ripper^41^ integrated a model of self-disclosure in the workplace and identified three ways individuals can control it: full disclosure, partial disclosure and non-disclosure.^42^,^46^ However, the existing studies did not address disclosure decisions specifically within the context of healthcare professionals, highlighting the need to gain more insight in this specific domain.

On an individual level, the phenomenon of disclosure can lead to a significant amount of stress owing to the pervasiveness of heteronormativity in healthcare workplaces, subjecting numerous LGBTQ+ staff to distressing microaggressions.^47^ These could include hurtful remarks, avoidance or discriminatory policies, all of which may gravely affect their mental well-being, particularly after identity disclosure, and may contribute to experiences of depression, anxiety and anger.^48 49^ On the other hand, the mental strain and the likelihood of developing depressive symptoms are both greater when one conceals their sexual orientation and/or gender identity.^1550^,^52^ Similarly, attempting to comply with societal norms can lead to similar adverse psychological impacts, including emotional fatigue; a major contributor to burnout.^53^

Although the individual effects of disclosure or nondisclosure are not unique to the healthcare sector, they pose an issue that calls for exploration through this specific lens, given the conventional expectations within the work environment.^7 13^ These risks have ripple effects across organisations. For instance, LGBTQ+ employees who cannot be their authentic selves may experience reduced social connections and job satisfaction,^53^ which, for some healthcare workers, has been linked to lesser productivity and engagement.^21^

Contrary to the potential negative consequences of identity concealment in the workspace, studies have shown positive outcomes of disclosure. Follmer *et al*,^14^ who reviewed 14 studies on workplace disclosure among LGBTQ+ staff, reported that visibility of one’s identity resulted in improved employee outcomes, encompassing enhanced job satisfaction, increased commitment to the organisation, and a notable reduction in job-related stress levels and turnover intentions. These findings encompassed not only LGBTQ+ workers but also a diverse range of stigmatised identities within the workplace. Further, a comprehensive study conducted by Ripper^41^ revealed that the theme of authenticity emerged as paramount among all participants. In this context, the ability to freely express oneself in a way that aligns with their identity, despite possible risks and fear of not being accepted, is seen as authentic.^17 54 55^ The study further highlighted that authenticity enhances interpersonal connections and strengthens the working experience, alongside serving as an essential phase in the process of workplace disclosure.^41^

Notably, the negative consequences often associated with disclosure can deter LGBTQ+ workers from revealing their identities.^20^ This suggests that the variables and outcomes of LGBTQ+ identity disclosure decisions in the workplace significantly influence each other. Therefore, it becomes crucial to recognise the significance of each disclosure decision and its unique manifestations in terms of risks and benefits.^11^

The literature discussed above offers an overview of workplace disclosure, focusing primarily on the experiences of LGBTQ+ workers.^14 53 56 57^ However, there remains a significant knowledge gap in comprehensively understanding LGBTQ+ identity disclosure in the workplace for healthcare professionals, as existing systematic reviews have mainly centred around patient perspectives or patient-provider disclosure.^858^,^60^ Moreover, the current literature has been predominantly qualitative and fragmented, highlighting the need for more quantitative research to explore the positive and negative outcomes of disclosure, and the variables that contribute to each, more thoroughly. Addressing this gap could lead to more inclusive and supportive work environments, benefiting both employees and organisations.

### The present study

While existing research recognises the importance of understanding LGBTQ+ workplace disclosure in healthcare settings, there remains a lack of common and shared meaning. This paper aims to fill this gap by exploring the experiences of disclosure in LGBTQ+ healthcare staff. The paper uses meta-synthesis to review the existing qualitative literature systematically using the thematic synthesis method and an integrative conceptual framework established from the works of McCormack *et al*^61^ and Peters *et al*.^62^

## Method

### Patient and public involvement

Patients and members of the public were not involved in the design of this study. The questions for this paper were generated in response to and in collaboration with an LGBTQ+ group of National Health Service (NHS) workers in the United Kingdom.

### Study design

A systematic review was conducted, evaluating primary studies which were selected based on the different steps outlined in the Cochrane handbook for conducting a systematic review.^63 64^ This was followed by a meta-synthesis. The chosen method was thematic synthesis, as described by Thomas and Harden.^65^ This research followed the guidelines outlined in the Preferred Reporting Items for Systematic Reviews and Meta-Analyses (PRISMA) 2020 statement^66^ and 27 items checklist for searching, selecting, evaluating and synthesising the studies, consequently enhancing transparency and validity to provide a complete unbalanced summary of evidence.

### Systematic review

#### Ethical considerations

This study implemented a secondary research design and constituted a comprehensive analysis of existing studies; hence, ethical clearance was not necessary. Nonetheless, the researchers demonstrated their commitment to ethical principles by handling the included studies responsibly. This was accomplished through the transparent presentation of the findings, thereby maintaining the integrity of the existing data and adhering to ethical standards in secondary research.

#### Eligibility criteria: inclusion and exclusion

Participants comprised individuals who identify as LGBTQ+ and work in healthcare settings. The PICoS (Population, Phenomenon of Interest, Context and Setting) method was applied to define inclusion and exclusion criteria.^67^ Experiences of disclosure were the exposure of interest.

The inclusion criteria included papers which were published, qualitative and peer-reviewed. We included countries that were United Nations members and had endorsed the LGBT rights declaration in the Human Rights Council in 2011. This ensured the clarity over the legal context and focused the analysis on variations in workplace disclosure experiences within states that recognised LGBT rights, excluding contexts where disclosure was criminalised. Consequently, the study’s eligibility criteria considered only papers published after 2011, ensuring relevance to the supportive statement signed by these nations.^68^ The studies which did not focus on experiences of disclosure between colleagues in the workplace, for example those that focused only on client and therapeutic interactions or healthcare professionals in training, were excluded from the search. Moreover, since the focus of the paper was to explore experiences, quantitative studies were excluded from the search. Finally, only studies published in English were selected.

#### Databases and information sources

The primary databases included PsycINFO (OVID); Journals@Ovid Full Text; Social Policy and Practice (OVID); PsycArticles (OVID). OVID served as the primary platform for conducting comprehensive tests and validation of distinct search queries, utilising various combinations of search terms and vocabulary.

Supplementary sources were comprehensively explored using a multi-faceted approach. This involved employing systematic hand searching techniques, as well as conducting forward and backward citation tracking of relevant papers. In addition, academic databases such as Google Scholar and Scopus were scanned to uncover valuable information and to expand the breadth of the research.

#### Search strategy: PICOS

Based on the research aim, a basic search string was created with the four categories of PICoS as discussed previously. The keywords were crafted by conducting a thorough review of pertinent literature and journal titles, with the aim of expanding the search scope and optimising the retrieval of relevant results. Nonetheless, the authors recognise the potential limitations in using certain terms, such as ‘non-heterosexual’, which might not be prevalent in LGBTQ+ literature. However, these terms were included in the search strings to align with the existing literature and ensure access to a diverse range of papers. Please refer to table 1 for a list of the keywords in the search terms and online supplemental material 1 for the full list of search strategies including the filters and databases.

**Table 1:** List of keywords and search terms using PICoS

PICoS category	Keywords
Population	sexual minority or lesbian or gay or bisexual or queer or non-heterosexual or LGBTQ or LGBTQ+or gender identity or gender expression or non-binary or sexual orientation or trans* or Homosexual or LGB*
Phenomenon of interest	coming out or outed or disclosure or non-disclosure or Self disclosure, disclosed, disclos* or com* out
Context	healthcare or NHS or hospital*
Setting	staff or occupational or employee* nurse* or doctor* or workplace or worker or professional

#### Selection and data extraction process

Following the steps specified by Ranganathan and Aggarwal,^69^ the first step involved screening abstracts of the papers retrieved from the databases. Owing to a high number of studies in the final search (n=4087 through Ovid), an additional screening step was implemented, involving a review of relevant papers based on their titles. As the search targeted only journal articles, there were no registers to consider. By employing EndNote and manual searching, 361 duplicates were removed.^70^ Additionally, seven studies were excluded before the screening process due to improper citations. A total of 3719 papers underwent title and abstract screening, during which 3670 papers were excluded based on the inclusion and exclusion criteria defined by PICoS. A language filter in Ovid to identify only English language papers published after 2011 proved effective and did not present any barriers, as these papers were not among those excluded in the screening phase. However, the research focus on LGBTQ+ healthcare professionals’ experiences resulted in a significant number of studies being filtered out, primarily due to their patient perspective or unrelated context.

Out of the remaining 49 studies, 7 were not accessible in full text. Consequently, in the second stage of full-text screening, 42 studies were identified with the consensus of four researchers (RP, LM, IA and SD). Out of these 42 studies, 37 had to be excluded, with 19 having an incorrect population focus (not exclusive to LGBTQ+ workers = 3, client-focused=16), 3 having an incorrect phenomenon of interest (unrelated to disclosure), 11 having an incorrect context (not focused on healthcare), and finally, 4 were not peer-reviewed studies. Similarly, studies were identified through other sources like Google Scholar and citation searching in a parallel manner. While acknowledging the ongoing debate over classifying Google Scholar as a website or database for systematic reviews, the author followed the conclusion of Boeker *et al*,^71^ considering it as a website source.

**Figure 1:**
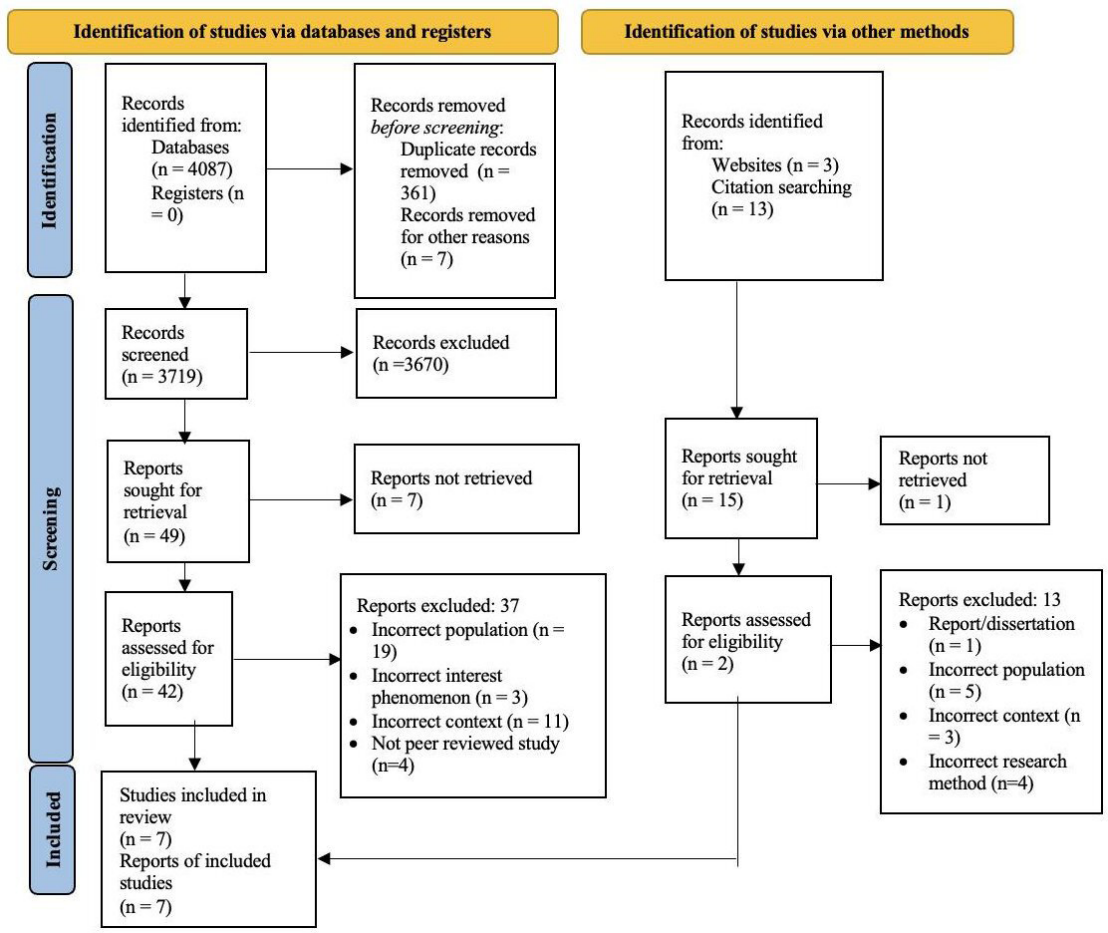
Preferred Reporting Items for Systematic Reviews and Meta-Analyses (PRISMA) flow chart. Overview of the search process using the PRISMA 2020 statement. Adapted from Page *et al*.^66^

A tabular summary of 21 papers was created after conducting a full-text assessment of 42 relevant papers. This comprehensive evaluation aided in the final selection of studies, considering participants, study design, disclosure decision, aims and summary. Any potentially divergent studies were discussed among the team using the table as a reference. Collaborative deliberations led all four researchers to reach a consensus and narrow down the choices to a refined set of papers deemed most suitable for the review. Please refer to figure 1 for a comprehensive overview of the search process, showcasing the PRISMA^66^ flow diagram. The figure provides a comprehensive visual representation of the search process, highlighting key numerical figures for each step and offering a detailed description of additional sources.

#### Quality assessment

The Critical Appraisal Skills Programme (CASP, 2022) checklist for qualitative studies, recommended by Cochrane Collaboration,^72^ was used to determine the quality across 10 questions. The assessment involved categorising responses to 10 questions as ‘yes’ if the information was presented, ‘no’ if it was missing, and ‘can’t tell’ if the information provided was insufficient for quality evaluation. Two independent reviewers conducted the quality appraisal, and consensus was reached through discussions among four authors.

### Synthesis measures

Applying the thematic synthesis method outlined by Thomas and Harden,^65^ it was decided that within the studies, the section labelled under ‘results’ would be considered for the data. This worked well for all studies, although ‘Older Lesbians and Work in the Australian Health and Aged Care Sector’^73^ lacked a distinct results section. Instead, the narratives and observations of the participants were considered for inclusion in the review.

During the process, two coders initially worked individually to enhance accuracy and reduce researcher bias, but later collaborated to validate the codes and themes. Coder one identified as cisgender and heterosexual, and coder two identified as cisgender and lesbian. As indicated by the literature^74 75^ and considering the qualitative nature, the researchers remained aware of their identities and perspectives throughout the coding process, recognising how these factors might influence the interpretation. The researchers did this by meeting regularly to talk through ideas, reflecting on the influence of their identities on the work and reflecting together about the process of defining themes and codes. The researchers chose to code on explicit rather than implicit contexts to reduce the imposition of researcher beliefs onto the text.

The synthesis process was carried out in three stages, involving continuous collaboration between the coders. The initial step focused on line-by-line coding of the results sections from various studies. While some studies explored general workplace experiences of LGBTQ+ healthcare workers, the coders agreed to concentrate specifically on experiences related to disclosure in the workplace. Additionally, disclosure with patients was coded, but the emphasis was on the overall experience and its effect rather than solely on the therapeutic relationship.

To facilitate the coding and tracking of categories, NVivo V.2020 software was employed, although the coding itself was also performed manually. Each of the seven papers was assigned an alphanumeric code in alphabetical order, enabling easy referencing to the original sources (table 3). The codes were centred around the main ideas presented in the sentences, highlighting various aspects of disclosure. Some codes were generated that may not have appeared directly relevant to the primary research question but were included to provide context. For instance, lines were also coded if they offered relevant explanations by the authors, even in the absence of direct quotations from the participants.

In the second step, the two coders cross-verified and grouped codes into 23 subthemes based on similarities. The exploration of themes in different articles was carried out, aligning the concepts in one article with the corresponding ideas in another, following current research practices.^76 77^ These subthemes were further classified where necessary.

The third and final step of the study identified five analytical themes, which were considered inferences at that stage. The themes were categorised into three distinct groups to facilitate better comprehension and classification based on the participants’ experiences, as discussed in the following section.

## Results

### Overview of studies

Seven papers were included in the thematic synthesis with four of them focusing on all healthcare professionals.^7 13 73 78^ The remaining three papers were dedicated to specific professions, namely physical therapists,^79^ radiation therapists^21^ and nurses.^80^ This paper included a total of 593 participants, comprising healthcare professionals from all 7 papers. The studies were conducted in various countries, with three in Canada, one in Australia, two in the USA and the last one spanning all three countries and the UK. The focus on workplace experiences of disclosure with both coworkers and patients was prevalent among all the papers.

The list of the included papers with the study characteristics is provided in table 2. Additionally, for reference, please refer to table 3 for the included paper titles and codes.

**Table 2:** Characteristics of included studies

Paper code	Author, year published	Participants’ information	Aim	Study design	Disclosure/non-disclosure to colleagues or patients
P1	Bizzeth and Beagan, 2023^78^	13 LGBTQ+ healthcare professionals, Canada	Explore work-related microaggressions and heteronormativity experiences	In-depth interviews	Both
P2	Ross *et al*, 2022^79^	22 LGBTQIA+ physical therapists, Australia, Canada, UK or USA	How physical therapists navigate their LGBTQIA+ identity in their professional roles	Semistructured interviews	Both
P3	Bolderston, 2021^21^	3 Lesbian Gay radiation therapists, Canada	Co-construct stories of coming out at work using their shared personal histories	Autoethnographic narrative approach	Both
P4	Eliason *et al*, 2018^7^	277 LGBTQ+ healthcare professionals, USA	Whether the coping strategies are specific to LGBTQ+ stress	Mixed-methods study- online survey +qualitative data	Both
P5	Eliason *et al*, 2011^80^	261 LGBTQ nurses, USA	Participants were asked how ‘out’ they were to friends, family, coworkers, employers and patients.	Open and closed ended online survey	Both
P6	Beagan *et al*, 2022^13^	13 LGBTQ+ healthcare professionals, Canada	Examine the experiences of health professionals (occupational therapists, nurses, physicians)	Semistructured interviews	Both
P7	Hughes and Kentlyn, 2015^73^	4 health or aged care workers, Australia	Participants’ reflection of their experience of work in the health and aged care sector, how this intersects with their identity(ies)	Semistructured interviews	Both

LGBTQ+, Lesbian, Gay, Bisexual, Transgender and Queer.

**Table 3:** List of included paper titles and codes

No.	Paper title	Paper code
1	“Ah, it’s best not to mention that here:” Experiences of LGBTQ+health professionals in (heteronormative) workplaces in Canada	P1
2	An Exploration of the Experiences of Physical Therapists Who Identify as LGBTQIA+: Navigating Sexual Orientation and Gender Identity in Clinical, Academic, and Professional Roles	P2
3	Coming out or staying in? Disclosure experiences of lesbian and gay radiation therapists in practice	P3
4	Coping With Stress as an LGBTQ+Health Care Professional	P4
5	Lesbian, Gay, Bisexual, Transgender, and Queer/Questioning Nurses’ Experiences in the Workplace	P5
6	LGBTQ+identity concealment and disclosure within the (heteronormative) health professions: “Do I? Do I not? And what are the potential consequences?”	P6
7	Older Lesbians and Work in the Australian Health and Aged Care Sector	P7

### Quality assessment

All seven final studies received approval across most of the 10 CASP^81^ questions, affirming the quality of the chosen papers (presented in online supplemental material 2). The table summarises evaluations of the seven selected papers using CASP, with all but two papers satisfying all 10 questions. Although Paper 2 lacked clear information on researcher relationships with participants, and Paper 7 presented unclear information on the process of data analysis, they were still included in the final review due to their ability to meet all the other criteria.

### Thematic synthesis

The thematic synthesis identified 3 categories, 5 themes and 23 subthemes. A summary of the identified categories, themes and their corresponding subthemes is presented in table 4. The detailed table containing the themes and corresponding codes with the paper codes for tracing and referencing is presented in online supplemental material 3. For a clear distinction, direct quotations from the participants have been indicated by quotes accompanying the codes, while the remaining codes represent the respective authors’ descriptions. In this section, each theme has been reported, followed by the presentation of overarching ideas through an integrative conceptual framework established from the works of McCormack *et al*^61^ and Peters *et al*.^62^

**Table 4:** Summary of identified categories, themes and subthemes

Category	Themes	Explanation of theme	Subthemes
Predisclosure	Disclosure carries risk	The potential risks associated and calculated assessments with disclosure.	Safety Risk to relationships at work Risk to professional progression/career Risk assessment and the mental toll
During disclosure	Making the decision to disclose (or not)	The process of contemplating whether to disclose after weighing the benefits and consequences.	Challenge or assimilate Influencing factors: trust, acceptance and judgement As a continuum Identity concealment Being outed Mental toll and discomfort
Effects of disclosure/non-disclosure	The cost of non-disclosure	The consequences and drawbacks of choosing not to disclose.	Lack of authenticity at work Hiding identity and mental toll Prevents individuals from challenging homophobia/transphobia Less able to connect with clients/colleagues
	The cost of disclosure	The negative repercussions and adverse outcomes of disclosure.	Professional cost Loss of relationship/distance with colleagues Loss of relationship/distance with clients Homophobia/transphobia/heterosexism
	The benefit of disclosure	The advantages and positive outcomes from making the decision to disclose.	Positive responses from colleagues Authenticity and connections Empowerment/challenging normative assumptions Therapeutic relationships are stronger Better connection with clients/colleagues

### Themes generated

#### Theme 1: disclosure carries risk

Risk assessment before disclosure was one of the most common themes echoed across all seven studies. Participants discussed weighing different outcomes in terms of risks to career and relationships at work. At times, it seemed like an inevitable decision to not disclose out of fear of the consequence, for instance, risk of safety or unpredictable responses of others:

Others simply never disclosed, because they could not predict responses: “I never know how they’re going to react …Would they be violent? Would they be aggressive? Would they report me to my boss or be super understanding?” (P6)

There were also instances where participants were outrightly warned^13^ or advised^7^ to not disclose their identities, shedding light on the lack of psychological safety at work:

Several people were warned by colleagues or superiors to be less open about their sexual identities: “It’s best not to mention that here.” (P6)

Risk relating to disclosure was often the result of heteronormative cis environments. Paper 2^80^ noted that while participants had differing narratives of the experiences, all were influenced by cisheteronormativity and the risks attached to defying it.

The process of selective disclosure relies on constantly assessing situations, calculating risk and benefit, plus the potential for disrupting normative expectations. (P1)

Paper 1^78^ highlighted an overlapping idea of disclosure and risk assessment of relationships with colleagues and patients. This paper tied together ideas of risks to relationships due to feeling othered by heteronormative assumptions and questions from coworkers and clients equally, rendering participants unsure whether to disclose or not. Additionally, Eliason *et al*^7^ reported religion playing a key role in workplace culture, with participants’ feeling unsafe about coming out among conservative, religious co-workers and risking the working relationships.

My immediate supervisor told me that he is a conservative Christian and that these ideals inspire him to make decisions at work. He also made disparaging comments about LGBTQ coworkers. This makes me feel threatened if I choose to be out at work. (P4)

A risk to professional or career progression was described by several participants across studies, with disclosure viewed as ‘unprofessional’ by others or a basis of judgement of professional ability.^73^ Participants feared disclosure presented a threat to their careers and a risk to their professional development as such. Participants reported undertaking a significant amount of ‘social calculations’ when considering disclosure.^79^ Most participants described the mental toll of this constant risk assessment.^79^

Early years in practice were marked with considerable energy devoted to deciding whether, when and how to disclose at work, performing careful risk assessments: “A lot of thought in disclosure, and more so, I guess, at the beginning, less so now… In the beginning, it was, I did find it stressful.” (P1)

#### Theme 2: making the decision to disclose (or not)

The authors of all seven papers identified the dilemma of whether to disclose LGBTQ+ identities in the workplace, with participants conflicted between disclosing and challenging assumptions of heteronormativity or concealing to avoid persecutory responses. Some individuals chose to disguise their LGBTQ+ identities to evade uncomfortable situations or discrimination.^13 21 73 78 79^ Conversely, there were those who opted to be open, refusing to hide and challenging prevailing heteronormative beliefs.^80^

Jan readily admitted that she was not open about her sexuality during her years as a medical specialist, although now she is an active member of a lesbian medical society. (P7)

Both trust and acceptance were the two key influencing factors in increasing participants’ sense of safety ahead of disclosure. Participants felt comfortable when trust was established with coworkers who encouraged them to not hide their LGBTQ+ identity. On the contrary, a fear of judgement and negative reactions deterred some from disclosing, causing distress:

Initially, John “wouldn’t elaborate,” describing the situation as “…quite a vulnerable spot to be in, to not know if you’re going to receive some judgement or negative response to finding out your sexuality.” (P2)

A key, recurring notion expressed across all papers was that disclosure is not a ‘single event’ located in time nor in relation to the specific parts of a person’s identity, but a ‘continuous and nuanced act’.^21^ The requirement to come out repeatedly and in multiple contexts across one’ career span was reported to carry a significant mental toll:

One participant described early years in clinical positions as “emotionally … exhausting, coming out over and over again.” (P6)

Owing to this mental toll, many participants described a fragmented sense of self and a significant impact on their mental health and well-being as a result:

…so split… In terms of different me’s… and who I could present them to. This [feeling] still has a residue of that sort of shame that I was carrying around for a long period of time… and it is part of my probably ongoing depression and anxiety. (P2)

Over time, many individuals selectively disclosed their identities to some colleagues and still did not disclose within new spaces, even if they were out with their coworkers.^13^ Some participants were open about certain aspects of their LGBTQ+ identity, like their sexuality, but kept other aspects, such as gender identity, private. Transgender participants encountered distinct challenges compared with LGB individuals, facing less acceptance and safety in the workplace.^7^

I’m currently out as queer to everyone and I am read as a woman married to a woman. I am not out as a trans man… I am worried about being out and transitioning… I am nervous that co-workers, supervisors, and patients will not want to interact with me. (P4)

Consequently, participants felt compelled to employ different strategies to conceal their LGBTQ+ identities in the workplace, including using gender-neutral language when referring to romantic partners or altering their appearance to avoid stereotypes. Many concealed their sexual orientation through vague responses and refrained from sharing specific details about their personal lives to limit disclosure.

In many instances, participants lost the power to make the decision to disclose for themselves by being ‘outed’ by colleagues. Some participants described a lack of support from relevant workplace systems following this experience, and even reported facing unfair dismissal from their role.^80^

#### Theme 3: the cost of non-disclosure

The concept of psychological strain was extended to the cost of non-disclosure, with Ross *et al*^79^ highlighting the ‘exhaustion’ in hiding one’ identity.

It was too bloody much work and it sucked…I don’t like hiding…. [in the end] I just wasn’t prepared to hide anymore because it was a lot of work…. (P2)

Three papers discussed the cost of not disclosing LGBTQ+ identities, with participants reporting a lack of authentic connections at work, and indeed a feeling of inauthenticity within themselves. Others felt it prevented them from challenging homophobia. Participants often experienced a barrier in relationships with others due to avoiding conversations with colleagues and feared a similar disconnect would hamper therapeutic rapports with clients.^13 79^

On the other hand, not “coming out” positions people as devious/inauthentic, and they experience ongoing fear about potential “discovery.” (P2)

#### Theme 4: the cost of disclosure

Themes of professional cost and loss of professional relationships as a result of disclosure were identified in five out of seven studies. Several participants described the microaggressions and adverse effects of workplace disclosure including harassment, gossip, hostility and loss of employment. Participants also expressed loss of relationships with both colleagues and clients, evident through their withdrawal and increased emotional distance. Cisheterosexism was also prevalent after disclosure, with participants reporting snide remarks and homophobic comments frequently, alongside microaggressions and assumptions surrounding LGBTQ+ community membership^73 78^:

In work contexts, participants found colleagues assumed all LGBTQ+people knew or could readily identify each other. The assumption that others can tell who is LGBTQ+again mobilizes stereotypes of LGBTQ+bodies and self-presentations, while simultaneously Othering LGBTQ+co-workers (P1)

#### Theme 5: the benefit of disclosure

Findings highlighted the positive effects of disclosure in the workplace such as positive responses from colleagues, empowerment and better connections. Indirectly, while working in cisheteronormative environments, individuals who were open about their LGBTQ+ identity helped promote acceptance and understanding among coworkers:

While everyone else that I work with is straight, and I think carry a fair amount of heterosexism, they are totally accepting of me and my family. For many of them, I am the only gay person they know, and I think that my being out and open about my life has helped them open their minds (P5)

Openness facilitated advocacy and enhanced rapport, establishing common ground and promoting inclusivity and connection with colleagues and clients. Finally, participants emphasised that disclosing their LGBTQ+ identity was crucial to their sense of self:

She reported that she feels that it’s crucial to her sense of self, makes no effort to hide it, and openly talks about her female partner. (P7)

### Presenting the thematic analysis using an integrative conceptual framework

**Figure 2:**
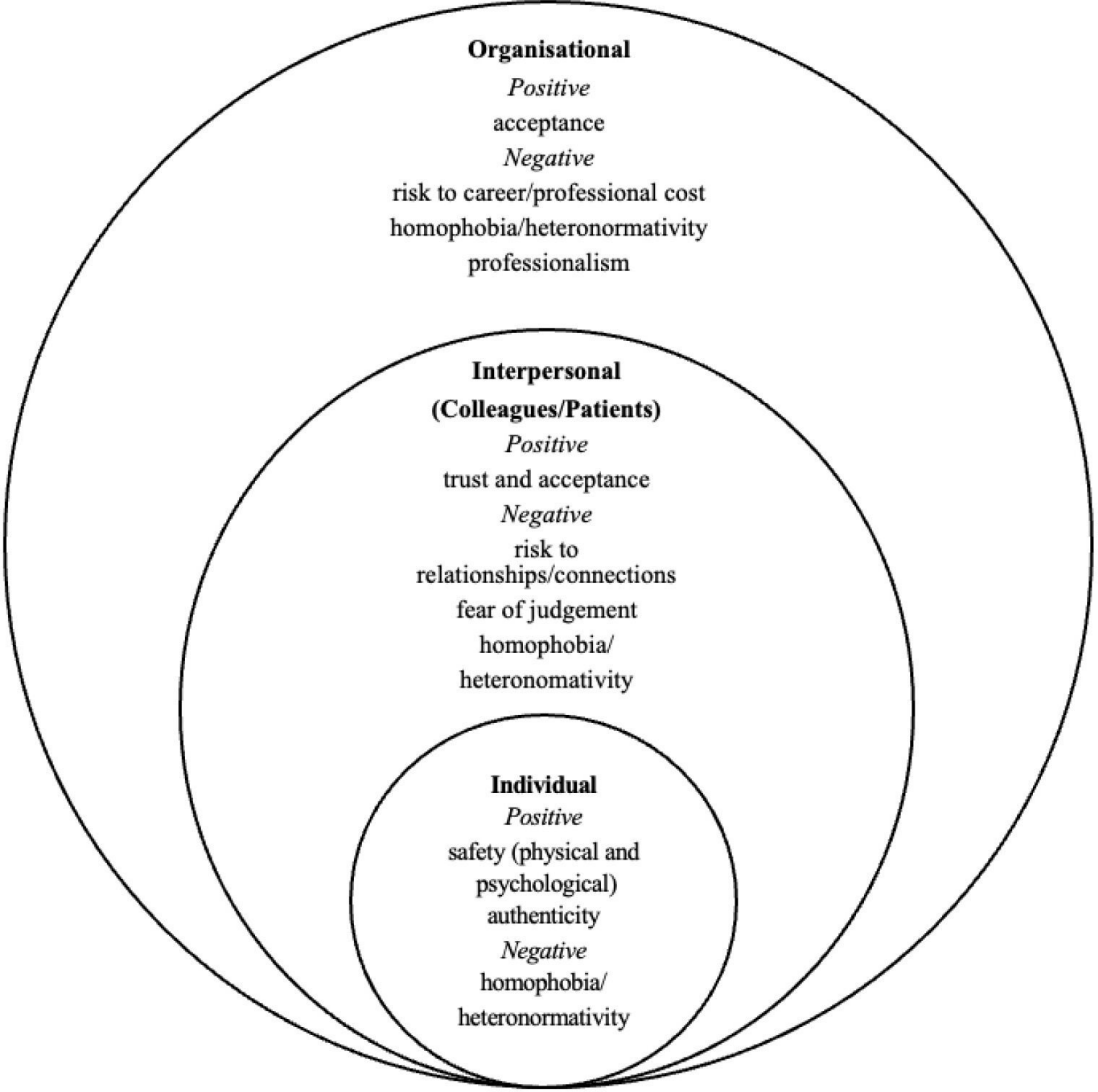
Integrated conceptual framework. An integrated conceptual framework was developed to identify positive and negative influencing factors towards disclosure in the workplace. All themes operated across different levels of the organisation.

All themes operated across different levels of the organisation. For instance, the most recurring themes explored authenticity at the individual level, connections and relationships at the group level, and acceptance at the organisational level (figure 2). The influencing factors displayed a similar pattern, exerting their impact on various groups, including co-workers and patients, thereby influencing the decision-making process indirectly. Consequently, even in cases where certain groups might not have directly affected the decision-making process, their involvement became apparent in the subsequent outcomes following disclosure.

Another concept widely mentioned across papers was disclosure as a continuum, in which the decision of making one’s stigmatised identity visible was often not a simple, one-time decision, but rather an ongoing process that occurred across different situations and contexts. Additionally, this exhaustive process also consumed mental energy of an individual, another common occurring subtheme. Overall, there was a delicate balance of disclosing and not disclosing the stigmatised identities within the workplaces. This was coupled with echoes of participants who usually found themselves grappling with this balance and tended to prioritise the comfort of others by not disclosing.

## Discussion

Based on the systematic review and meta-synthesis of seven qualitative papers with five prominent themes (table 4), it was found that heteronormativity and microaggressions existed throughout different levels (figure 2) and guided disclosure decisions. The study also developed two important aspects of disclosure: that it exists on a continuum; and places a mental toll on the individual both before and throughout the process.

### Summary and interpretation of results

In accordance with current literature, the identified themes suggest that workplace disclosure is a dynamic process.^20 27 56 57 82^ While earlier reviews on general workplace disclosure offered valuable insights into aspects such as time and context,^14 57^ this review paper focused specifically on healthcare settings and expanded the understanding of distinct variables throughout the disclosure process. Three additional themes were identified, totalling five, where disclosure could differ based on an employee’s perception of the work environment. These variations could occur individually or simultaneously.

Participants across the studies revealed that the level of disclosure could be full, partial or null, with the null level indicating complete absence or non-disclosure of one’s LGBTQ+ identity.^13 78^ The scope of disclosure also varied, with some individuals coming out to their close colleagues and others only disclosing to select patients to enhance therapeutic rapport.^13 80^ Often, this was influenced by time, with many participants gradually revealing their LGBTQ+ identity.^21 79^ It was not always necessary for all aspects of identity to be revealed during the process of disclosure, with many choosing to disclose certain elements, such as their gender identity or sexual orientation only.^7 79^ Finally, LGBTQ+ identity was disclosed through different methods, with some openly discussing it from the start of their employment, some choosing to reveal their identity to educate people against homophobia, and others choosing to come out during one-to-one conversations with co-workers.^13 79^

These findings suggest that concealing one’s queer identity, particularly within the healthcare sector, may be necessary to avoid homophobic behaviours and unwanted reactions from colleagues and patients. This aligns with previous quantitative research,^20^ reporting that concealment may be an inevitable choice in a hostile or unsupportive environment, rather than a personal decision and a reflection of the final stage of LGBTQ+ identity development.

In contrast, participants who described having supportive colleagues reported fewer concerns around disclosure and disclosed their queer identities more often than their counterparts who lacked a supportive team, providing an insight into perceived co-worker support. Finally, two consistent factors across all stages and most narratives of disclosure were the prioritisation of others’ comfort, such as colleagues or patients, over one’s own, and the mental toll of hiding or constantly risking revealing one’s identity in the workplace. This may be interpreted as a cause-and-effect scenario, with the former leading to distress or psychological strain when LGBTQ+ employees choose not to disclose at work.

The theme of the benefits of disclosure yielded only nine codes. There are a few potential explanations for this finding. The first may be a reflection of participants’ limited experience of workplace disclosure, possibly as a reflection of associated negative connotations and perceptions. The second, although disclosure of one’ LGBTQ+ identity may enhance feelings of connection and authenticity in some, for others, perhaps disclosure itself does not carry additional benefits, simply the alleviation of the mental toll of hiding a part of one’s identity. Finally, it is possible that current qualitative research has focused on the negative impacts of and barriers to disclosure and has not extensively examined the potential benefits of disclosure.

### Implications

#### Theoretical

The presented integrated conceptual framework classified the variables into three levels: interpersonal, intrapersonal and institutional, in line with previous literature.^83 84^ A notable factor across all three levels was heteronormativity, which significantly influenced workplace disclosure. Participants felt constrained by the prevailing discourses of ‘professionalism’, fearing that being open about their identities as healthcare staff might be considered unprofessional. This finding aligns with existing literature, which describes ‘heteroprofessionalism’ as the phenomenon wherein sexuality and/or gender identity is viewed as inappropriate within the context of professionalism, particularly for identities that deviate from normative expectations.^78 84 85^

#### Methodological

In contrast to some studies, our established PICoS framework encompassed a diverse range of healthcare professionals by considering variables like employment position, age and ethnicity for the possible scope for intersectionality. This method may have implications for how intersectionality will be explored in field research in the future. The internal experiences of LGBTQ+ healthcare workers in negotiating their personal identities within the professional sphere can be inferred from the findings obtained from this meta-synthesis.

#### Applied

Presently, there are more guidelines in place for healthcare workers to care for LGBTQ+ patients than for supporting the professionals themselves.^86^,^88^ Such findings are not surprising when considered in conjunction with results from a recent survey conducted by the British Medical Association (BMA) and Association of LGBTQ+ Doctors and Dentists (GLADD), that reports 71% of lesbian, gay, bisexual and queer respondents report biphobia and homophobia to be an ongoing issue in medicine, with 43% experiencing direct homophobia or biphobia at least once in the last year.^4^ Similar findings were reported by Eliason *et al*,^89^ who found that 15% of LGBT physicians in the USA reported harassment by coworkers and 22% experienced social ostracism at work.

Despite laws and declarations endorsing LGBTQ+ rights, healthcare workers still face discrimination based on their identity.^68^ This ranges from being explicitly asked to conceal their identities at work to experiencing denial of or delayed promotions. A report by the Stonewall Charity in the UK revealed that many LGB professionals choose not to disclose their identities due to discrimination within their workplace, raising a need to reevaluate the current policies, practices and guidelines.^90^

### Strengths, limitations and future recommendations

#### Strengths

The use of systematic and rigorous tools, such as the Cochrane handbook, PRISMA 2020 statement and 27 items checklist, to find, assess and report qualitative research is a strength.^63 66^ Additionally, CASP questionnaires tailored to study design were used to assess quality and provide some uniformity in the questions concerning bias assessment across research. To further our understanding, we also included narratives and direct quotes from the studies where the authors’ analyses included workplace disclosure experiences.

By using the PICoS method, this study ensured a comprehensive examination of LGBTQ+ staff experiences through qualitative studies, considering the diverse factors that might influence their experiences.^67^ Therefore, this study included individuals based on factors such as job position, age and ethnicity, to acknowledge and explore the potential intersections among these variables. This approach allowed for a nuanced understanding of how LGBTQ+ staff navigated their identity in the healthcare workplace, considering potential challenges and opportunities that may have arisen due to intersectionality.

#### Limitations

The limitations of this paper are divided into two broad categories: the limitations in the review process and limitations in evidence.

In any qualitative review study, the limitations in the review process become evident, particularly concerning errors in overlooking relevant literature and considering researcher positionality. For instance, when interpreting the theme of responsibility of empowerment, its positive or negative connotation was ambiguous. Based on the nature of the statement, our inference leant towards a positive interpretation, emphasising how a researcher’s perspective can influence the results.

All the studies were based in countries with a predominant sample of Western, Educated, Industrialised, Rich and Democratic populations.^91^ While this research provides insight into the workplace situation in those regions, it lacks representation of cultural differences and developing countries, resulting in a lack of generalisability. Similarly, while there was an effort to include all demographics, only one study yielded a connection between age and LGBTQ+ identity in the workplace.^73^ Additionally, as the review included in-depth qualitative extraction and thematic synthesis, only English-language publications were included to ensure consistent coding and interpretation. This language limitation may have resulted in the exclusion of relevant non-English publications.

### Recommendations for future

For future analyses, novel research exploring workplace disclosure experiences and challenges in countries where LGBTQ+ identities are not legalised is recommended. For instance, Brooks *et al*’s^92^ review highlighted how criminalisation in certain countries posed an additional barrier to workplace disclosure from a patient perspective, emphasising the need for more focused research.

We identified a gap in research, with only one paper acknowledging the intersectionality of LGBTQ+ identity and age.^73^ Future research can explore intersectionality since individuals’ numerous, intertwined social identities operate alongside frameworks of power and privilege to create systemic disadvantages. Thus, it becomes essential to theorise through this lens when analysing the communication of LGBTQ+ employees, recognising that various subgroups within the community may experience disclosure differently. Investigations into sexual orientation and gender should be connected with experiences related to ethnicity, disability, religion, nationality and age since these issues are entwined,^17 93 94^ all of which have been found to affect employee performance, employee satisfaction and ultimately staff well-being.^95^ Researchers should also explore the content and construct validity for workplace disclosure. Future studies must specifically show uniformity and exclude the use of outdated terminology.^96^

In line with previous research,^17 41^ it was found that creating a healthy work environment that is supportive of all can foster feelings of safety and comfort in the workplace, thus enabling professionals to consider whether to disclose their LGBTQ+ identity without fear of negative repercussions. Building a positive work community that promotes diversity through fostering relationships, teamwork and collaboration, with visible acceptance of colleagues who choose to disclose their LGBTQ+ identities, can also minimise the barriers to disclosure and support queer healthcare professionals to bring as much of their identity to the workplace as they so choose.

### Conclusions

This research presents the first qualitative meta-synthesis to explore the disclosure experiences of LGBTQ+ healthcare workers. We found that cis-heteronormative practices dominate the working environment of healthcare staff. This often resulted in non-disclosure, as employees sought to assimilate and protect themselves from the consequences of non-inclusive workplaces. In particular, the study highlights that the decision to disclose is influenced by various factors across different levels, and the process of disclosure manifests on a continuum. Our findings reveal mixed experiences of workplace disclosure, and how it subsequently impacts the mental well-being of employees and organisational structures themselves. As a result, LGBTQ+ healthcare workers have unique organisational communication concerns at work. Together, the findings presented in this paper demonstrate the importance of fostering a psychologically safe and meaningfully inclusive workplace within which LGBTQ+ employees are able to bring their full selves without fear, should they wish to do so.

## Supplementary material

10.1136/bmjopen-2025-100412 online supplemental file 1

10.1136/bmjopen-2025-100412 online supplemental file 2

10.1136/bmjopen-2025-100412 online supplemental file 3

## Data Availability

All data relevant to the study are included in the article or uploaded as supplementary information.

## References

[1] Mohanty A, Kabi A, Mohanty AP (2019). Health problems in healthcare workers: A review. J Family Med Prim Care.

[2] Romate J, Rajkumar E (2022). Exploring the experiences, psychological well-being and needs of frontline healthcare workers of government hospitals in India: a qualitative study. *Humanit Soc Sci Commun*.

[3] Hoel H, Lewis D, Einarsdottir A (2014). The ups and downs of LGBs’ workplace experiences: discrimination, bullying and harassment of lesbian, gay and bisexual employees in Britain.

[4] Sexual orientation and gender identity in the medical profession.

[5] Rhead RD, Chui Z, Bakolis I (2021). Impact of workplace discrimination and harassment among National Health Service staff working in London trusts: results from the TIDES study. BJPsych open.

[6] Stonewall (2020). List of LGBTQ+ terms.

[7] Eliason MJ, Streed C, Henne M (2018). Coping With Stress as an LGBTQ+ Health Care Professional. J Homosex.

[8] Meads C, Hunt R, Martin A (2019). A Systematic Review of Sexual Minority Women’s Experiences of Health Care in the UK. Int J Environ Res Public Health.

[9] Blondeel K, Say L, Chou D (2016). Evidence and knowledge gaps on the disease burden in sexual and gender minorities: a review of systematic reviews. Int J Equity Health.

[10] Owens B, Mills S, Lewis N (2022). Work-related stressors and mental health among LGBTQ workers: Results from a cross-sectional survey. PLoS One.

[11] Chrobot-Mason D, Button SB, DiClementi JD (2001). Sexual Identity Management Strategies: An Exploration of Antecedents and Consequences. Sex Roles.

[12] Berkley RA, Beard R, Daus CS (2019). The emotional context of disclosing a concealable stigmatized identity: A conceptual model. Human Resource Management Review.

[13] Beagan BL, Bizzeth SR, Pride TM (2022). LGBTQ+ identity concealment and disclosure within the (heteronormative) health professions: “Do I? Do I not? And what are the potential consequences?”. SSM - Qualit Res Health.

[14] Follmer KB, Sabat IE, Siuta RL (2020). Disclosure of stigmatized identities at work: An interdisciplinary review and agenda for future research. J Organ Behavior.

[15] Witte TK, Kramper S, Carmichael KP (2020). A survey of negative mental health outcomes, workplace and school climate, and identity disclosure for lesbian, gay, bisexual, transgender, queer, questioning, and asexual veterinary professionals and students in the United States and United Kingdom. J Am Vet Med Assoc.

[16] Thuillier J, Almudever B, Croity-Belz S (2022). Perceived Workplace Discrimination and Disclosure at Work Among Lesbian and Gay Employees: The Role of Prior Coming Out Experiences in Different Life Domains. J Homosex.

[17] Holmberg MH, Martin SG, Lunn MR (2022). Supporting sexual and gender minority health-care workers. Nat Rev Nephrol.

[18] van der Toorn J, Pliskin R, Morgenroth T (2020). Not quite over the rainbow: the unrelenting and insidious nature of heteronormative ideology. Curr Opin Behav Sci.

[19] Brady B, Asquith NL, Ferfolja T (2022). Fear of Heterosexism Among Sexuality and Gender Diverse Staff and Students. J Interpers Violence.

[20] Ragins BR, Singh R, Cornwell JM (2007). Making the invisible visible: fear and disclosure of sexual orientation at work. J Appl Psychol.

[21] Bolderston A (2021). Coming out or staying in? Disclosure experiences of lesbian and gay radiation therapists in practice. Radiography (Lond).

[22] Emetu RE, Rivera G (2018). After Sexual Identity Disclosure: An Ecological Perceptive of LGB Young Adults. Am J Health Behav.

[23] Maliepaard E (2018). Disclosing Bisexuality or Coming Out? Two Different Realities for Bisexual People in The Netherlands. J Bisex.

[24] Collins NL, Miller LC (1994). Self-disclosure and liking: a meta-analytic review. Psychol Bull.

[25] Griffith KH, Hebl MR (2002). The disclosure dilemma for gay men and lesbians: “coming out” at work. J Appl Psychol.

[26] Mosher CM (2001). The Social Implications of Sexual Identity Formation and the Coming-Out Process: A Review of the Theoretical and Empirical Literature. Family J.

[27] Gray EM (2013). Coming out as a lesbian, gay or bisexual teacher: negotiating private and professional worlds. Sex Educ.

[28] Goldberg AE The sage encyclopedia of lgbtq studies, 2455 Teller Road, Thousand Oaks, California 91320.

[29] Knous HM (2006). The coming out experience for bisexuals: Identity formation and stigma management. J Bisex.

[30] Ragins BR (2008). Disclosure Disconnects: Antecedents and Consequences of Disclosing Invisible Stigmas across Life Domains. AMR.

[31] Turner JC, Oakes PJ (1986). The significance of the social identity concept for social psychology with reference to individualism, interactionism and social influence. British J Social Psychol.

[32] Turner JC (2010). Towards a cognitive redefinition of the social group. In Research Colloquium on Social Identity of the European Laboratory of Social Psychology, Dec, 1978, Université de Haute Bretagne, Rennes, France; This chapter is a revised version of a paper first presented at the aforementioned colloquium.

[33] Altymurat A, Muhai M, Saparow T (2021). Human Behavior in Organizations Related to Abraham Maslow’s Hierarchy of Needs Theory. *IJPHR*.

[34] Maslow AH (1958). A dynamic theory of human motivation.

[35] Thalberg I, Maslow AH (1964). Toward a Psychology of Being. Philos Phenomenol Res.

[36] Anzalotta J (2017). I am human, too! An analysis of conflict resolution theories and their applicability to the LGBTQ community.

[37] Brooks-Jones F Strategies early childhood elementary teachers use in order to provide a welcoming environment for students who they perceive to be lgbtq or exhibit stereotypical characteristics. Doctoral dissertation.

[38] Hutton SL Understanding school climate and interventions for lesbian, gay, bisexual, transgender, and questioning students. Doctoral dissertation.

[39] Haslanger S (2023). Systemic and Structural Injustice: Is There a Difference?. Philosophy.

[40] Jackson M Responsibilities for reform: essays on addressing systemic injustice. Master’s thesis.

[41] Ripper M (2021). A multiple case study exploration of resilience, disclosure, and workplace behavior in LGBTQ employees.

[42] Chaudoir SR, Fisher JD (2010). The disclosure processes model: understanding disclosure decision making and postdisclosure outcomes among people living with a concealable stigmatized identity. Psychol Bull.

[43] Griffin P (1992). From hiding out to coming out: empowering lesbian and gay educators. J Homosex.

[44] Huang HY (2016). Examining the beneficial effects of individual’s self-disclosure on the social network site. Comput Human Behav.

[45] Lewis GB (1995). The Corporate Closet: The Professional Lives of Gay Men in America. Public Adm Rev.

[46] Wang L, Yan J, Lin J (2017). Let the users tell the truth: Self-disclosure intention and self-disclosure honesty in mobile social networking. Int J Inf Manage.

[47] Nadal KL (2008). Preventing racial, ethnic, gender, sexual minority, disability, and religious microaggressions: Recommendations for promoting positive mental health. Prev Couns Psychol.

[48] Resnick CA, Galupo MP (2019). Assessing Experiences With LGBT Microaggressions in the Workplace: Development and Validation of the Microaggression Experiences at Work Scale. J Homosex.

[49] Vaccaro A, Koob RM (2019). A Critical and Intersectional Model of LGBTQ Microaggressions: Toward a More Comprehensive Understanding. J Homosex.

[50] Madera JM (2010). The Cognitive Effects of Hiding One’s Homosexuality in the Workplace. Ind Organ Psychol.

[51] Sedlovskaya A, Purdie-Vaughns V, Eibach RP (2013). Internalizing the closet: concealment heightens the cognitive distinction between public and private selves. J Pers Soc Psychol.

[52] Spiegel T, De Bel V, Steverink N (2016). Keeping up appearances: the role of identity concealment in the workplace among adults with degenerative eye conditions and its relationship with wellbeing and career outcomes. Disabil Rehabil.

[53] Webster JR, Adams GA, Maranto CL (2018). Workplace contextual supports for LGBT employees: A review, meta‐analysis, and agenda for future research. Hum Resour Manage.

[54] Authenticity HS (2002). Handbook of positive psychology.

[55] Riggle EDB, Mohr JJ, Rostosky SS (2014). A multifactor Lesbian, Gay, and Bisexual Positive Identity Measure (LGB-PIM). Psychol Sex Orientat Gend Divers.

[56] Croteau JM, Anderson MZ, VanderWal BL (2008). Models of workplace sexual identity disclosure and management: Reviewing and extending concepts. Group & Organization Management.

[57] Wax A, Coletti KK, Ogaz JW (2018). The benefit of full disclosure: A meta-analysis of the implications of coming out at work. Organizat Psych Rev.

[58] Dahl B, Fylkesnes AM, Sørlie V (2013). Lesbian women’s experiences with healthcare providers in the birthing context: a meta-ethnography. Midwifery.

[59] Harding R, Epiphaniou E, Chidgey-Clark J (2012). Needs, Experiences, and Preferences of Sexual Minorities for End-of-Life Care and Palliative Care: A Systematic Review. J Palliat Med.

[60] St. Pierre M (2012). Under What Conditions do Lesbians Disclose Their Sexual Orientation to Primary Healthcare Providers? A Review of the Literature. J Lesbian Stud.

[61] McCormack B, Karlsson B, Dewing J (2010). Exploring person-centredness: a qualitative meta-synthesis of four studies. Scand J Caring Sci.

[62] Peters L, Hobson CW, Samuel V (2022). A systematic review and meta-synthesis of qualitative studies that investigate the emotional experiences of staff working in homeless settings. Health Soc Care Community.

[63] Higgins JPT, Thomas J, Chandler J (2022). Cochrane Handbook for Systematic Reviews of Interventions version 6.3 (updated February 2022). www.training.cochrane.org/handbook.

[64] Linares-Espinós E, Hernández V, Domínguez-Escrig JL (2018). Methodology of a systematic review. *Actas Urológicas Españolas (English Edition*).

[65] Thomas J, Harden A (2008). Methods for the thematic synthesis of qualitative research in systematic reviews. BMC Med Res Methodol.

[66] Page MJ, McKenzie JE, Bossuyt PM (2021). The PRISMA 2020 statement: an updated guideline for reporting systematic reviews. BMJ.

[67] Amir-Behghadami M, Janati A (2020). Population, Intervention, Comparison, Outcomes and Study (PICOS) design as a framework to formulate eligibility criteria in systematic reviews. Emerg Med J.

[68] US Mission to International Organizations in Geneva (2011). Over 80 Nations Support Statement at Human Rights Council on LGBT Rights.

[69] Ranganathan P, Aggarwal R (2020). Study designs: Part 7 - Systematic reviews. Perspect Clin Res.

[70] (2017). EndNote. https://endnote.com/.

[71] Boeker M, Vach W, Motschall E (2013). Google Scholar as replacement for systematic literature searches: good relative recall and precision are not enough. BMC Med Res Methodol.

[72] Noyes J, Popay J, Pearson A (2008). Cochrane handbook for systematic reviews of interventions: cochrane book series.

[73] Hughes M, Kentlyn S (2015). Older lesbians and work in the Australian health and aged care sector. J Lesbian Stud.

[74] Bleiker J, Morgan-Trimmer S, Knapp K (2019). Navigating the maze: Qualitative research methodologies and their philosophical foundations. Radiography (Lond).

[75] Thorne S (2000). Data analysis in qualitative research. Evid Based Nurs.

[76] Lachal J, Revah-Levy A, Orri M (2017). Metasynthesis: An Original Method to Synthesize Qualitative Literature in Psychiatry. Front Psychiatry.

[77] Noblit GW (1988). Meta-ethnography: synthesizing qualitative studies.

[78] Bizzeth SR, Beagan BL (2023). “Ah, it’s best not to mention that here:” Experiences of LGBTQ+ health professionals in (heteronormative) workplaces in Canada. Front Sociol.

[79] Ross MH, Hammond J, Bezner J (2022). An Exploration of the Experiences of Physical Therapists Who Identify as LGBTQIA+: Navigating Sexual Orientation and Gender Identity in Clinical, Academic, and Professional Roles. Phys Ther.

[80] Eliason MJ, Dejoseph J, Dibble S (2011). Lesbian, gay, bisexual, transgender, and queer/questioning nurses’ experiences in the workplace. J Prof Nurs.

[81] Critical Appraisal Skills Programme (2022). Critical Appraisal Checklists. https://casp-uk.net/casp-tools-checklists/.

[82] Orne J (2011). ‘You will always have to “out” yourself’: Reconsidering coming out through strategic outness. Sexualities.

[83] Paisley V, Tayar M (2016). Lesbian, gay, bisexual and transgender (LGBT) expatriates: an intersectionality perspective. Int J Human Resource Manag.

[84] Calvard T, O’Toole M, Hardwick H (2020). Rainbow Lanyards: Bisexuality, Queering and the Corporatisation of LGBT Inclusion. Work, Employment Soc.

[85] Mizzi RC (2013). “There aren’t any gays here”: encountering heteroprofessionalism in an international development workplace. J Homosex.

[86] Eliason MJ, Chinn P, Dibble SL (2013). Open the door for LGBTQ patients. Nursing (Auckl).

[87] McNair RP, Hegarty K (2010). Guidelines for the primary care of lesbian, gay, and bisexual people: a systematic review. Ann Fam Med.

[88] Williams H, Varney J, Taylor J (2013). The lesbian, gay, bisexual and trans public health outcomes framework companion document. Public Health England.

[89] Eliason MJ, Dibble SL, Robertson PA (2011). Lesbian, gay, bisexual, and transgender (LGBT) physicians’ experiences in the workplace. J Homosex.

[90] Hunt R, Cowan K, Chamberlain B (2007). Being the gay one: experiences of lesbian, gay and bisexual people working in the health and social care sector.

[91] Henrich J, Heine SJ, Norenzayan A (2010). The weirdest people in the world?. Behav Brain Sci.

[92] Brooks H, Llewellyn CD, Nadarzynski T (2018). Sexual orientation disclosure in health care: a systematic review. Br J Gen Pract.

[93] Cyrus K (2017). Multiple minorities as multiply marginalized: Applying the minority stress theory to LGBTQ people of color. J Gay Lesbian Ment Health.

[94] Eger EK, Litrenta ML, Kane SR (2022). LGBTQ+ workers. InOxford research encyclopedia of communication 2022 May 18.

[95] Woodhead C, Stoll N, Harwood H (2022). “They created a team of almost entirely the people who work and are like them”: A qualitative study of organisational culture and racialised inequalities among healthcare staff. Sociol Health Illness.

[96] Robertson IT, Cooper CL, Sarkar M (2015). Resilience training in the workplace from 2003 to 2014: A systematic review. J Occupat & Organ Psyc.

