# Supplementary material for: Disclosure experiences in LGBTQ+ healthcare staff: a systematic review and meta-synthesis
Source: BMJ Open. 2026 Mar 4;16(3):e100412. doi: 10.1136/bmjopen-2025-100412 (PMC12970120; doi:10.1136/bmjopen-2025-100412)
Supplement: online supplemental file 1 [file bmjopen-16-3-s001.docx]

**Supplemental Material 1: Literature Search and Search Strings**

Ovid – search

Journals@Ovid Full Text < March 31, 2023>

APA PsycArticles Full Text

APA PsycInfo <1806 to July Week 2 2023>

Social Policy and Practice <202304>

Your Journals@Ovid

1 LGBTQ+.mp. [mp=tx, bt, ti, ab, hw, tn, ot, dm, mf, dv, kf, fx, dq, nm, ox, px, rx, an, ui, ds, on, sy, ux, mx, cw, ct, sh, tc, id, tm, pt] 15826

2 sexual minority.mp. [mp=tx, bt, ti, ab, hw, tn, ot, dm, mf, dv, kf, fx, dq, nm, ox, px, rx, an, ui, ds, on, sy, ux, mx, cw, ct, sh, tc, id, tm, pt] 22996

3 lesbian.mp. [mp=tx, bt, ti, ab, hw, tn, ot, dm, mf, dv, kf, fx, dq, nm, ox, px, rx, an, ui, ds, on, sy, ux, mx, cw, ct, sh, tc, id, tm, pt] 63682

4 gay.mp. [mp=tx, bt, ti, ab, hw, tn, ot, dm, mf, dv, kf, fx, dq, nm, ox, px, rx, an, ui, ds, on, sy, ux, mx, cw, ct, sh, tc, id, tm, pt] 114632

5 bisexual.mp. [mp=tx, bt, ti, ab, hw, tn, ot, dm, mf, dv, kf, fx, dq, nm, ox, px, rx, an, ui, ds, on, sy, ux, mx, cw, ct, sh, tc, id, tm, pt] 75387

6 trans*.mp. [mp=tx, bt, ti, ab, hw, tn, ot, dm, mf, dv, kf, fx, dq, nm, ox, px, rx, an, ui, ds, on, sy, ux, mx, cw, ct, sh, tc, id, tm, pt] 69187

7 queer.mp. [mp=tx, bt, ti, ab, hw, tn, ot, dm, mf, dv, kf, fx, dq, nm, ox, px, rx, an, ui, ds, on, sy, ux, mx, cw, ct, sh, tc, id, tm, pt] 20889

8 non-heterosexual.mp. [mp=tx, bt, ti, ab, hw, tn, ot, dm, mf, dv, kf, fx, dq, nm, ox, px, rx, an, ui, ds, on, sy, ux, mx, cw, ct, sh, tc, id, tm, pt] 2389

9 LGBT*.mp. [mp=tx, bt, ti, ab, hw, tn, ot, dm, mf, dv, kf, fx, dq, nm, ox, px, rx, an, ui, ds, on, sy, ux, mx, cw, ct, sh, tc, id, tm, pt] 34831

10 gender identity.mp. [mp=tx, bt, ti, ab, hw, tn, ot, dm, mf, dv, kf, fx, dq, nm, ox, px, rx, an, ui, ds, on, sy, ux, mx, cw, ct, sh, tc, id, tm, pt] 92478

11 gender expression.mp. [mp=tx, bt, ti, ab, hw, tn, ot, dm, mf, dv, kf, fx, dq, nm, ox, px, rx, an, ui, ds, on, sy, ux, mx, cw, ct, sh, tc, id, tm, pt] 3528

12 non-binary.mp. [mp=tx, bt, ti, ab, hw, tn, ot, dm, mf, dv, kf, fx, dq, nm, ox, px, rx, an, ui, ds, on, sy, ux, mx, cw, ct, sh, tc, id, tm, pt] 4815

13 sexual orientation.mp. [mp=tx, bt, ti, ab, hw, tn, ot, dm, mf, dv, kf, fx, dq, nm, ox, px, rx, an, ui, ds, on, sy, ux, mx, cw, ct, sh, tc, id, tm, pt] 76578

14 1 or 2 or 3 or 4 or 5 or 6 or 7 or 8 or 9 or 10 or 11 or 12 or 13 300837

15 Coming out.mp. [mp=tx, bt, ti, ab, hw, tn, ot, dm, mf, dv, kf, fx, dq, nm, ox, px, rx, an, ui, ds, on, sy, ux, mx, cw, ct, sh, tc, id, tm, pt] 302336

16 disclosure.mp. [mp=tx, bt, ti, ab, hw, tn, ot, dm, mf, dv, kf, fx, dq, nm, ox, px, rx, an, ui, ds, on, sy, ux, mx, cw, ct, sh, tc, id, tm, pt] 986156

17 out*.mp. [mp=tx, bt, ti, ab, hw, tn, ot, dm, mf, dv, kf, fx, dq, nm, ox, px, rx, an, ui, ds, on, sy, ux, mx, cw, ct, sh, tc, id, tm, pt] 21900593

18 non-disclosure.mp. [mp=tx, bt, ti, ab, hw, tn, ot, dm, mf, dv, kf, fx, dq, nm, ox, px, rx, an, ui, ds, on, sy, ux, mx, cw, ct, sh, tc, id, tm, pt] 4943

19 minority stress.mp. [mp=tx, bt, ti, ab, hw, tn, ot, dm, mf, dv, kf, fx, dq, nm, ox, px, rx, an, ui, ds, on, sy, ux, mx, cw, ct, sh, tc, id, tm, pt] 9404

20 experiences.mp. [mp=tx, bt, ti, ab, hw, tn, ot, dm, mf, dv, kf, fx, dq, nm, ox, px, rx, an, ui, ds, on, sy, ux, mx, cw, ct, sh, tc, id, tm, pt] 1813865

21 Healthcare staff.mp. [mp=tx, bt, ti, ab, hw, tn, ot, dm, mf, dv, kf, fx, dq, nm, ox, px, rx, an, ui, ds, on, sy, ux, mx, cw, ct, sh, tc, id, tm, pt] 20595

22 NHS.mp. [mp=tx, bt, ti, ab, hw, tn, ot, dm, mf, dv, kf, fx, dq, nm, ox, px, rx, an, ui, ds, on, sy, ux, mx, cw, ct, sh, tc, id, tm, pt] 433211

23 occupational.mp. [mp=tx, bt, ti, ab, hw, tn, ot, dm, mf, dv, kf, fx, dq, nm, ox, px, rx, an, ui, ds, on, sy, ux, mx, cw, ct, sh, tc, id, tm, pt] 1234965

24 healthcare workforce.mp. [mp=tx, bt, ti, ab, hw, tn, ot, dm, mf, dv, kf, fx, dq, nm, ox, px, rx, an, ui, ds, on, sy, ux, mx, cw, ct, sh, tc, id, tm, pt] 7382

25 healthcare workplace.mp. [mp=tx, bt, ti, ab, hw, tn, ot, dm, mf, dv, kf, fx, dq, nm, ox, px, rx, an, ui, ds, on, sy, ux, mx, cw, ct, sh, tc, id, tm, pt] 883

26 healthcare worker*.mp. [mp=tx, bt, ti, ab, hw, tn, ot, dm, mf, dv, kf, fx, dq, nm, ox, px, rx, an, ui, ds, on, sy, ux, mx, cw, ct, sh, tc, id, tm, pt] 109460

27 health provider*.mp. [mp=tx, bt, ti, ab, hw, tn, ot, dm, mf, dv, kf, fx, dq, nm, ox, px, rx, an, ui, ds, on, sy, ux, mx, cw, ct, sh, tc, id, tm, pt] 73548

28 health professional*.mp. [mp=tx, bt, ti, ab, hw, tn, ot, dm, mf, dv, kf, fx, dq, nm, ox, px, rx, an, ui, ds, on, sy, ux, mx, cw, ct, sh, tc, id, tm, pt] 487436

29 15 or 16 or 17 or 18 or 19 or 20 23250832

30 21 or 22 or 23 or 24 or 25 or 26 or 27 or 28 2222663

31 14 and 29 and 30 5683

32 limit 31 to english 5682

33 limit 32 to transgender 5640

34 limit 33 to yr="2011 -Current" 4087
