# Supplementary material for: Disclosure experiences in LGBTQ+ healthcare staff: a systematic review and meta-synthesis
Source: BMJ Open. 2026 Mar 4;16(3):e100412. doi: 10.1136/bmjopen-2025-100412 (PMC12970120; doi:10.1136/bmjopen-2025-100412)
Supplement: online supplemental file 2 [file bmjopen-16-3-s002.docx]

**Supplemental Material 2: CASP Checklist**

*Critical Appraisal Skills Programme (CASP) Checklist for included studies*

| **Paper Code** | **Q1** | **Q2** | **Q3** | **Q4** | **Q5** | **Q6** | **Q7** | **Q8** | **Q9** | **Q10** |
| --- | --- | --- | --- | --- | --- | --- | --- | --- | --- | --- |
| P2 | yes | yes | yes | yes | yes | can’t tell | yes | yes | yes | yes |
| P1 | yes | yes | yes | yes | yes | yes | yes | yes | yes | yes |
| P3 | yes | yes | yes | yes | yes | yes | yes | yes | yes | yes |
| P5 | yes | yes | yes | yes | yes | yes | yes | yes | yes | yes |
| P6 | yes | yes | yes | yes | yes | yes | yes | yes | yes | yes |
| P4 | yes | yes | yes | yes | yes | no | yes | yes | yes | yes |
| P7 | yes | yes | yes | yes | yes | no | yes | can’t tell | yes | yes |

*Note.* Adapted from Programme, C. A. S. (2022). CASP Systematic Review Checklist. <https://casp-uk.net/casp-tools-checklists/>

1. Questions included - 1. Was there a clear statement of the aims of the research? 2. Is a qualitative methodology appropriate? 3. Was the research design appropriate to address the aims of the research? 4. Was the recruitment strategy appropriate to the aims of the research? 5. Was the data collected in a way that addressed the research issue? 6. Has the relationship between researcher and participants been adequately considered? 7. Have ethical issues been taken into consideration? 8. Was the data analysis sufficiently rigorous? 9. Is there a clear statement of findings? 10. How valuable is the research?
