# Supplementary material for: Disclosure experiences in LGBTQ+ healthcare staff: a systematic review and meta-synthesis
Source: BMJ Open. 2026 Mar 4;16(3):e100412. doi: 10.1136/bmjopen-2025-100412 (PMC12970120; doi:10.1136/bmjopen-2025-100412)
Supplement: online supplemental file 3 [file bmjopen-16-3-s003.docx]

**Supplemental Material 3: Themes & Codes**

| **S.No.** | **Sub-Theme** | **Code and Quotation** |
| --- | --- | --- |
| **THEME 1: DISCLOSURE CARRIES RISK** | | |
| 1 | Safety | - lack of safety   - As one participant said, “Students that are not out don't tend to come out during their [professional education]. They're afraid of what might happen. So there's still an abiding fear within even the younger generations.” (P1) - This participant “shut the door” on conversations about families and relationships, fearing for her safety if she disclosed LGBTQ+ identity; yet she worried keeping things superficial harmed her therapeutic work. (P1) - Some watched for conversational “red ﬂags” signalling a patient might be unsafe to disclose to, aiming to avoid being “hurt in some way.” (P6) - Others simply never disclosed, because they could not predict responses: “I never know how they're going to react …Would they be violent? Would they be aggressive? Would they report me to my boss or be super understanding?” (P6)   - warnings/cautions not to disclose   - In their professions, and in work contexts, participants were often advised not to disclose LGBTQ+ identities. One participant said she was warned by an older LGBTQ+ colleague, “Be careful who you tell.” Another was warned “to be more quiet about it”: “just saying like you better not talk about that, so-and-so staff member is really uncomfortable with that and may treat you differently.” (P1) - This was echoed by another participant: “I actually had one of the preceptors [clinical educators] tell me—I said something about my partner and the preceptor actually said to me, ‘Ah, it's best not to mention that here.”' (P1) - On a more pronounced level, health care providers within mental health care settings stated that they were advised not to be out to patients because of the unpredictable nature of psychiatric patients. “I work in mental health, so self-disclosure is not appropriate,” stated one of the respondents. (P4) - “Yes and no, it's an environment of don't ask don't tell.” (P5) - Several people were warned by colleagues or superiors to be less open about their sexual identities: “It's best not to mention that here.” (P6)   - experiences of homophobia / heteronormativity   - Participants felt pressured to make complex disclosure decisions in the face of such heteronormative questions and assumptions. (P1) - Yet people had to actively decide how to respond to the heteronormative assumptions of coworkers and patients. As Stenger and Roulet found in their study of auditors, LGBTQ+ professionals engaged in “shamming, distance, and normification” (P1) - The process of selective disclosure relies on constantly assessing situations, calculating risk and benefit, plus the potential for disrupting normative expectations. (P1) - The pressure to conform to a cis-/hetero-normative physical therapy environment often positions participants in dilemmas of “choice” (often spanning many years, colleagues, workplaces, and patients) about disclosing (or not) their gender identity or sexual identity. Participants indicate that neither option is without stress. (P2) - Although participants had quite different stories about this, with some hiding their sexual orientation and others being open about it, all were shaped by discourses of hetero-normativity. (P2) |
| 2 | Risk to relationships at work | - fear of judgement   - Being open about LGBTQIA+ identity places people at risk of feeling “different” or “odd” and potentially facing discrimination. (P2)   - religion   - one respondent was not out because “I have two very conservative Christian coworkers with a lot of power and they already look at me with some suspicion because I’m not religious.” (P4) - Working in an environment with religiously conservative coworkers was associated with negative experiences for many LGBTQ+ health care providers. The remarks made by religiously conservative staff members can cause fear in coming out at work. Exemplifying this, a respondent recollected: “My immediate supervisor told me that he is a conservative Christian and that these ideals inspire him to make decisions at work. He also made disparaging comments about LGBTQ coworkers. This makes me feel threatened if I choose to be out at work.” (P4) - Commonly, the reason for the non-LGBTQ+-friendly workplace environment was a conservative religious climate: “My clinic is pretty conservative and lots of their staff members are fundamentalist Christians. I am out to my good friends, but not to all. There is a lot of overt talk about God and religion at work. But of the people who know, they are all fine with me.”(P4) - Participant 32, a bisexual female, said that the environment was unfriendly because it was “very religious. Catholic organization. No visibility whatsoever.” (P5)   -with colleagues   - one participant described feeling Othered when in casual conversations with professional colleagues, never fitting their expectations but fearing talking about her life would label her as deviant: “You know, you go have a cup of tea or something with them, and it's like, you know, some of the questions: Okay, well, where do you work? Where do you live? What's your family? Do you have a family? Do you have a husband? Right? It's always that sort of, “Do you have a husband?” normative questions, always.” (P1) - Although participants responded in different ways to this positioning, almost all participants describe commonly feeling the need to conceal their sexual orientation, or aspects of it, among colleagues (P2)   -with clients   - As one lesbian health professional described, “Patients who are like, ‘Oh, do you have a boyfriend?' sometimes I'll say ‘No, I have a girlfriend,' and other times I don't, and it's a judgement that I make. Sometimes, you have to err on the side of caution.” As one participant argued, it is important to avoid “potentially opening yourself up to a whole world of hurt.” (P1) - Yet, some people were concerned that distancing to avoid disclosure or heterosexist microaggressions could also hinder their ability to build rapport with patients: “You build a rapport with them over time. But, it's sort of like, I would always shut that door very, very quickly. And yeah, I feel like it doesn't allow for as natural an exchange as, say I had a husband… It seems almost easier to build rapport with clients when you're of that normative kind of status.” (P1) - “I think “being out” is a slippery concept in my professional life. I am out as both queer and trans/genderqueer as a general rule, in all aspects of my life, but when I am working with patients I am more focused on them and what they need than on myself and who I am or am not. Sometimes disclosing more about myself improves care and sometimes it is irrelevant. Sometimes patients treat me with respect and sometimes they don’t.” (P4) - A trend within the theme of discrimination occurred for some physicians and mental health care providers who stated feeling uneasy being out for risk of making the patient feel uncomfortable and losing them as a patient. (P4) - “I'm always sort of conscious and aware of that when I'm speaking with clients, and trying to gauge honestly whether or not they would accept me, if I was open to them about my identity.” (P6) |
| 3 | Risk to professional progression / career | -disclosure as unprofessional   - There was a general sense that disclosing LGBTQ+ identity was inappropriate in professional contexts: “It was a professional environment in the sense that it just never came up. I don't know how to say it. It just never came up. Like, with my colleagues, it was always about work. (P1) - This was particularly strong regarding disclosing to clients; as one participant commented, “it's a professional boundary, right? I think for most people your first ‘go to' is going to be not disclosing too much.” (P1) - “With patients? No, it doesn't come up. Why should it? Like I said, I think we should be professional. There is a line, I don't like to cross it.” (P3) - Reasons for not being out were generally related to fears of losing the job or facing potential harassment and discrimination. (P4)   - judgement of professional ability   - One participant suggested disclosing sexual identity in the context of hands-on care for patients was inherently risky: “You would be potentially opening yourself up to a whole world of hurt, and possible allegations of misbehaving or of inappropriate conduct… It's boundaries. And you just don't want to put yourself or your colleagues in jeopardy.” (P6) - Another person worried professional credibility would be undermined with disclosure: “With clients, I would worry that they would think less of my capability, you know, trust me less, just have an opinion of some sort that could potentially be negative.” (P6)   - professional risk/ capacity to work   - They describe how these “social calculations” affect their capacity to work as a physical therapist daily by consuming cognitive and emotional energy. (P2) - “perception that if people knew I am a lesbian they would not want me examining their children.” (P5)   - focus on sexuality by others   - Some participants felt tokenized, reduced to their queerness, with workplace colleagues employing nicknames like “Team Rainbow” that focused on their sexual identities. This focus, or even unwanted “outing,” could be uncomfortable, even when intended light-heartedly (P1) - She also fiercely resisted the whole idea of “coming out.” In terms of her working life, Thelma said that her sexuality has never been a problem because she never spoke about it. Indeed, for Thelma, her sexuality was not only something that should not be disclosed, but also something that should not be conceptualized or labeled. (P7) |
| 4 | Risk assessment and the mental toll | - Early years in practice were marked with considerable energy devoted to deciding whether, when and how to disclose at work, performing careful risk assessments: “A lot of thought in disclosure, and more so, I guess, at the beginning, less so now… In the beginning, it was, I did find it stressful.” (P1) - Discourses of stress were evident in all stories often related to multi-level lack of safety, including fearing discrimination for expressing sexual orientation or gender identity, ongoing challenges of repeatedly coming out (or not) in cis/hetero-normative environments, and the effects of these stressors on people’s health. (P2) - Some participants describe the serious and enduring impact these stressors have on their mental health. For example, Nathan describes feeling: “…so split… In terms of different me’s… and who I could present them to. This [feeling] still has a residue of that sort of shame that I was carrying around for a long period of time. I think it has still fed into, and it is part of my probably ongoing depression and anxiety that I still have.” (P2) - Having to make this choice results in considerable inner conflict for participants. Participant Ellie calls it “doing the safety math.” (P2) - Risk was assessed on multiple levels; in discussing reasons for identity concealment, one person said, “I don't think I would have been unsafe, like, physically unsafe. Certainly emotionally, possibly somewhat unsafe.” (P6) - Another detailed the anxiety that disclosure induced: “I would describe it as stressful … A lot of thought on disclosure, and more so, I guess, at the beginning, less so now. But, always an underlying thought of ‘Do I? Do I not? And what are the potential consequences?’ Whether it was with clients or whether it was with coworkers or whether–” (P6) |
| **THEME 2: MAKING THE DECISION TO DISCLOSE (OR NOT)** | | |
| 1 | Challenge or assimilate | - talking about deciding   - Sometimes—depending on their assessment of the situation, as well as their own energy to engage—people actively misrepresented their LGBTQ+ identities, as described by a gay man: [Patients] would say “Are you in a relationship?” and I would say “Yes,” and they would then say, “Oh, what does your girlfriend do?” and I would just be like, “Oh, she's an engineer.” Just to avoid the conversation, honestly, because in some instances… in a busy day, when I don't want to have an uncomfortable interaction, it felt easier to just lie.” (P1) - only one participant was out throughout their professional education, in every professional context, including with clients/patients: “I'm not hidden. I've been out for the last 25 plus years. I refuse to hide, go back in the closet.” That person had been out for about a decade before entering professional education. (P6) - Jan readily admitted that she was not open about her sexuality during her years as a medical specialist, although now she is an active member of a lesbian medical society. (P7)   - challenge assumptions of heteronormativity - choosing to challenge or assimilate   - “I was working as a circulating nurse and the anesthesiologist and surgeon were making negative comments on gay marriage. I came out to them and let them know how inappropriate their comments were and was mocked later in the case...the man they were operating on happened to be gay. When I brought this to the attention of my supervisor, I was not encouraged to pursue any action.” (P5) - “I never said anything about LGBT discriminatory things … I don’t think I ever had the courage to do so, because I think, for me, defending it is an admission of being–… like if I stand up for LGBTQ rights, then to that patient, at least to them, I am probably disclosing myself as a member of that community, and then putting myself at risk for that, or for further discrimination which at that point in my career, I didn’t want to bear the brunt of.” (P6) |
| 2 | Influencing factors: trust, acceptance, and judgement | - disclosure requires trust? to people you trust   - Occasionally, when participants felt safe enough to always be “out” at work, they found this enhanced connection with LGBTQ+ patients, facilitating common ground and stronger rapport. (P1) - Participant disclosure of sexual orientation, when it occurred, was enabled by specific contexts (eg, HIV rehabilitation) or after developing trust over a series of interactions. (P2) - The worry associated with being out to more than trusted coworkers caused feelings of discomfort, and participants often noted feeling threatened. (P4) - In recent years, she did not hide her sexual identity in the workplace, but she did only want to tell people if they became friends. Some people knew, some guessed: “the word was around.”(P7)   - acceptance by others   - John describes how visible acceptance of other “out” classmates and colleagues made him feel safe and confident to come out in both university and the workplace. (P2) - “In general people are very nice at my workplace about it [coming out]. I have never heard anyone say anything negative about queer people.” (P4)   - fear of judgement   - Due to a fear of judgment, Samira describes recent experiences of coming out in her current workplace as “horrible...this feeling in the pit of my stomach, like a sick sensation. I'd Get Clammy. I’d feel really hot, like that feeling before you're about to faint or something....” (P2) - Initially, John “wouldn’t elaborate,” describing the situation as “…quite a vulnerable spot to be in, to not know if you’re going to receive some judgement or negative response to finding out your sexuality.” (P2) |
| 3 | As a continuum | - ongoing process   - Making LGBTQ+ identities evident is not a single event; it entails a continuum from complete disclosure to complete non-disclosure (P1) - Participants described a variety of ways in which they were open about their sexual orientation: by starting to correct people when they made mistakes, by making time to “come out” in one-on-one discussions with colleagues they trusted, and by bringing up their sexual orientation from the very start of their employment. (P2) - Listening to my participants' stories (and telling my own), I heard several repetitive ideas about coming out. For example, ‘let people come out in their own time’ and ‘coming out is a continuous and nuanced act’. I realised I had internalised certain “rules” about coming out that I had never really articulated but were attached to strong social mores. (P3) - “Also I tend to go on and off masculinizing hormones, so I never know how my patients perceive my gender and by extension my sexuality. When I mention my partner (also genderqueer) and use male pronouns, I don’t know if I am perceived as straight or gay. It varies from moment to moment. I see up to 20 patients a day and sometimes they’re all new patients and I have zero idea how I will be perceived/received as I move from room to room. It can be stressful”. (P4) - “While everyone else that I work with is straight, and I think carry a fair amount of heterosexism, they are totally accepting of me and my family. For many of them, I am the only gay person they know, and I think that my being out and open about my life has helped them open their minds” (P5) - Most often, people had moved from greater to lesser identity concealment over time, often engaging in selective disclosure. One participant, asked about being out during school, replied, “Um – (laugh) – to some. Not exactly, I wouldn't say. I mean, it kind of gradually, you know, it takes a while for me sometimes, when I'm meeting people, to gain trust. But over the years, I'm more out now.” (P6) - Participants were most likely to disclose queer identities to colleagues, typically increasingly so over time. Some described speaking openly about partners, using gendered pronouns, bringing partners to work-related social events, having partners drop by for lunch at work, and having photos of partners/families in their work spaces. One participant described organizing events for LGBTQ þ Pride as a student, and one had challenged professors on heterosexism. (P6)   - coming out/concealment in multiple spaces   - Some had been more “out” before entering their professions, then “chose to be more and more closeted” as they progressed in their fields. (P1) - Three others were open about their sexual identities throughout their education and careers, though usually not with clients/ patients. Everyone else concealed their queer identities at least some times and/or at particular career stages. (P6) - Some had been out before professional education but began concealing identities in what they described as a conservative environment: “In the pre-clinical years, my classmates knew my partner. But I think I actually chose to be more and more closeted, (laugh) throughout the clinical years.” As one participant said, “Students that are not out don't tend to come out…They're afraid of what might happen. So there's still an abiding fear within even the younger generations.” (P6) - Even those who were out with all of their usual colleagues had moments where concealment/disclosure arose again: “Professionally, I run workshops, I speak at conferences, and you know, I think we all naturally speak about the people that are close to us in our lives. But I do kind of pause and, and I don’t know why that is.” (P6) - two participants did not disclose their LGBTQ þ identities at all with their colleagues, opting to pass as straight. One person was only out with non-work friends: “No. (laugh) Yeah, no, like, it's honestly, it's really only friends. It's not something I bring with me to work … I've never brought it up, ‘cause, again, it's a weird thing to talk about generally in the workplace.” (P6) - Three others disclosed selectively. For example, one participant disclosed with a few very close work colleagues, but not more generally: “No, no, because it was a professional environment in the sense that it just never came up. I don't know how to say it. It just never came up. With my colleagues, it was always about work. None of my personal stuff.” (P6)   - being out about certain elements of identity eg sexuality but not others eg. gender identity   - Even if participants are “out” to some or all of the people in their work environment, there are often parts of their LGBTQIA+ lives that are important to them they do not feel are acceptable to discuss at work. (P2) - Another respondent also had more anxiety about their transgender identity than their sexual identity and stated: “I’m currently out as queer to everyone and I am read as a woman married to a woman. I am not out as a trans man to anyone beyond my spouse, counselor, and a few close friends. Being queer and masculine of center has generally been okay although I find that I am more cautious in situations because I am not sure if people will find me threatening. I am worried about being out and transitioning as a trans man and I am nervous that coworkers, supervisors, and patients will not want to interact with me.” (P4) - One respondent who did not identify on the trans spectrum confirmed that the climate is different for transgender people than for LGB providers (P4) - “Trans folks are a whole other issue. I don’t identify as trans but all the tolerance people have managed to develop around the choice to be gay has not been extended to trans people in the same way. There are a lot of gay people in my work place so if anyone has homophobic attitudes it is not a safe environment to express them.” (P4) |
| 4 | Identity concealment | - covering   - passing (Goffman, 1963), distancing from others, and covering—striving to render queerness less objectionable to avoid stigma (Yoshino, 2006). Our participants did the same. (P1) - Passing can entail avoiding disclosure, but can also mean outright misdirection. As one participant said about responding to patient questions, “There would be many times that I'd just say, “Oh, I'm not married” or that sort of thing. I wouldn't give away anything more than that. And sometimes, I wouldn't even say anything. I would just kind of smile and nod and deflect.”(P1) - One participant described his response as “declining to elaborate:” “I even sometimes find myself still kind of—not lying, but maybe sometimes still hiding, or you know just declining to disclose or elaborate.” (P1) - John describes how he deflected questions to hide his sexual orientation. When patients ask questions such as: “Do you have a girlfriend?” John mentions, at times, he has responded: “No, I don’t have a girlfriend.” (P2) - Some of them sometimes ask about my wife, or girlfriend but I just like to say partner. I don't think it is appropriate to ever really get into anything more specific than me having a partner with patients. (P3) - To avoid unwanted disclosures, several participants used impression management strategies, particularly covering. For example, some women were careful to dress and style their hair to avoid looking “too butch.” (P6) - Some gay man altered their bodily comportment and their clothing selection to avoid stereotyped flamboyance: “I would wear bright clothing in my personal life, but I don't think I'd do it very much in my work. Maybe a pink shirt I might wear now. But that's taken a long time to do that.” (P6)   - hiding behind heteronormativity   - Many participants discussed feeling the need to hide their sexual orientation in professional contexts. For example, Charlie said: “It was [difficult]. But it didn't seem to be difficult at the time, you just kept your mouth shut ... and whenever I needed to [make it appear that I was heterosexual], I could always say, "Oh, well my kids." Blah, blah, blah. Or, "My husband." That immediately puts out any fire or flames, or anything that people maybe think might be there, that knocks that on the head, and they go, "Oh, okay, good, you're okay then." … I was going to cop [take] it. If I didn't cop it for being gay, I was going to cop it for doing it to the kids.’ (P2) - Charlie’s story indicates that being non-heterosexual is positioned in physical therapy as outside of the norm. As a result, Charlie describes taking quite extreme measures to hide her relationships with women. (P2) - “Some of them sometimes ask about my wife, or girlfriend but I just like to say partner. I don't think it is appropriate to ever really get into anything more specific than me having a partner with patients.” (P3) - Even a participant who had organized medical students walking in the Pride parade, described sometimes “hiding” queer identity: “In clerkship … through rotations, I definitely was hiding myself in certain instances and I would think about whether or not I could disclose, which in itself is a problem, right? You shouldn’t be shy about mentioning the fact that you have a partner, to anyone and everyone, especially when you’re talking about ‘Hey what are you doing this weekend?’ But in medical school, yes, I was kind of at times hiding.” (P6) |
| 5 | Being outed | - lost power to make the decision   - “I was “outed” by a person I supervised, and it resulted in having to go to HR [human resources]. HR said I couldn’t be “harassed” because sexual harassment doesn’t happen to men.” (P4) - A few reported serious consequences from being “outed,” for example, a few nurses reported job loss after years of stellar reviews. (P5) - “Being outed by a colleague at a faculty party. Although all of my evaluations had been excellent and I had just completed my master's I did not have my contract renewed. I had been teaching in the program for 9 years. It may have had nothing to do with me being gay but it was awfully coincidental.” (P5) |
| 6 | Mental toll and discomfort | - “I keep to myself. Very closeted. Scared to talk about it.” (P5) - One participant described early years in clinical positions as “emotionally … exhausting, coming out over and over again.” (P6) - Continuing, the participant reflected, “That bothers me, to be honest. I don't like that's how I feel. I'd like to get to where I don't kind of pause for thought or speak about her differently than I would, were she male.” (P6) - Angela said she is cautious about who she discloses her sexuality to, as much for their comfort as for her own. She reported that her experiences have been colored, to a certain degree, by having been sent to a psychologist when she was young to be treated for her sexuality. (P7) |
| **THEME 3: THE COST OF NON-DISCLOSURE** | | |
| 1 | Lack of authenticity at work | - lack of authenticity at work   - On the other hand, not “coming out” positions people as devious/inauthentic, and they experience ongoing fear about potential “discovery.”(P2)   - hiding identity   - Participant Rahul uses the metaphor “turn[ing] my light down” to describe the impact of hiding his sexual orientation and other marginalized characteristics to fit into constrained ideas of professional norms in physical therapy. (P2) |
| 2 | Hiding identity and mental toll | - Many participants discuss stressful experiences of discrimination or fear of discrimination. For example, Mary shares her early experiences among colleagues: “I was completely closeted and terrified that my physio classmates would find out because a number of them were assholes and made really homophobic comments....” (P2) - Similarly, Mary discusses the exhaustion she felt being closeted during her physical therapy career in the United States: “It was too bloody much work and it sucked...I Don't Like Hiding...[in the end] I just wasn’t prepared to hide anymore because it was a lot of work....” These stories demonstrate the considerable labor involved in hiding sexual orientation and gender identity and other risks of disclosure. (P2) |
| 3 | Prevents individual from challenging homophobia / transphobia | - When not overtly disclosing LGBTQ+ identity, confronting heterosexist microaggressions risks eliciting “guilt by association,” potentially incurring stigma. (P1) |
| 4 | Less able to connect with clients / colleagues | - One way of avoiding disclosures was detachment (Stenger and Roulet, 2018). One participant commented, “[I] found myself kind of avoiding talking about my personal life in a variety of situations,” including with patients and coworkers. (P1) - Another had ceased engaging with colleagues socially, growing tired of dodging questions and comments that assumed heterosexuality: “So, I tend to avoid social situations as best as I can.” (P1) - People described keeping engagement with clients/patients superﬁcial: “No, I deﬁnitely don't share much with my clients, just surface things.” (P6) - One participant questioned whether identity concealment harmed therapeutic rapport; a barrier is erected by not being able to engage in everyday chatter with clients/patients about families, weekend activities and such.. (P6) - “...I think, conﬂicts with that therapeutic rapport that you’re supposed to be able to establish with your patients, because you’re always hiding something. There’s certain parts of your life that they might want access to, that you just don’t want to open up to.” (P6) |
| **THEME 4: THE COST OF DISCLOSURE** | | |
| 1 | Professional cost | - “I hate to say it because I don't think this is the best, but I think good advice might be to actually be more quiet… Sometimes being different can actually work quite against you… It's just not a culture that wants to promote diversity… If the people in power don't like that, then they can kind of um, maybe make you in a position where you are more likely to be ostracized.” (P1) - The type of discrimination took several forms, including coworkers using inappropriate gender pronouns, refusal of tenure, harassment, loss of patients, and delay of academic promotion. (P4) - One physician explained, “As a sole provider, the only problem I feel I sometimes face is discrimination from potential patients as they are selecting a health care provider.” (P4) - Worrying about the ramifications of being out at work was the second most prevalent theme in the findings, occurring in 27% of the respondents who acknowledged this has led to workplace problems.(P4) - One respondent recalled, “In my previous job, I had many challenges. Gossip behind my back, challenges to why I was teaching about LGBT issues, a refusal of tenure (overturned by administration), and lots of hostility.(P4) - “I lost my job after posting my wedding in the local paper, after over a decade at the same job. Never underestimate the power of a Catholic hospital.” (P5) - She was also concerned that it could have had an adverse effect on her career. She recounted a problem with a colleague but he was a “nasty customer” who would use anything to advance his career and grab power and seems to have thought her sexuality was a weapon he could use against her. (P7) |
| 2 | Loss of relationship/ distance with colleagues | - Some reported that when colleagues knew their LGBTQ+ identities, it seemed to stifle the usual co-worker chat that can lubricate workplace interactions: “We had a good working relationship, but they never asked me about my personal life, as they would the other colleagues… It's like, just missing out on some of those social conversations.” (P1) - With colleagues, heterosexist assumptions left participants caught between invisibility and unwanted hyper-visibility. (P1) - As another participant described, disclosure often disrupted connection: “It's probably only when you experience these things is when you notice it, but the pause or the facial expression is different…” In heteronormative work contexts, casual chatter about LGBTQ+ lives could hinder connection, but so could avoiding casual conversations. (P1) - A few participants were out about their sexuality but were afraid that coworkers and patients would have a harder time understanding and accepting their gender identities. (P4) - Some had colleagues withdraw, become remote, or give them “weird looks” when the participant's sexual identity became known. (P6) |
| 3 | Loss of relationship/ distance with clients | - At the same time some participants found disclosing LGBTQ+ identities could also harm rapport, causing patients/clients to distance: “I've never had an experience where they were overtly homophobic. It's more people would stop opening up to me, or they would become suddenly very awkward and standoffish, and my rapport with them changed.” (P1) - Another participant acknowledged it was always hard to tell if patient withdrawal after disclosure was due to stigma and discrimination, or simply processing unexpected information. (P6) |
| 4 | Homophobia / transphobia / heterosexism | - In work contexts, participants found colleagues assumed all LGBTQ+ people knew or could readily identify each other. The assumption that others can tell who is LGBTQ+ again mobilizes stereotypes of LGBTQ+ bodies and self-presentations, while simultaneously Othering LGBTQ+ co-workers (P1) - Of the 62% who were out, a perception of being discriminated against was the most pronounced theme in the findings, occurring in 41% of the respondents who acknowledged that being out has caused problems in the workplace. (P4) - She said her main concern in “coming out” to colleagues was to prevent these “really nice people” from “falling into traps of making, you know, homophobic comments, that sort of thing.” They were also “really against PhDs and doctors, and I had to say, ‘Well, look, I’m a PhD’. Yeah, so I was lesbian and PhD. Double whammy.” (P7) - Angela reported mixed responses to her sexuality by colleagues in the workplace. Early in her career, “some of the guys … made some snide remarks about it once they found out,” although other colleagues such as psychologists, social workers, doctors, and psychiatrists were more accepting and supportive. However, she was acutely aware that, at that time, “homosexuality was still a disease. We came out at a time where … making love to a person of your own sex was certainly not an accepted thing. So that influences even today.” (P7) |
| **THEME 5: THE BENEFIT OF DISCLOSURE** | | |
| 1 | Positive responses from colleagues | - “While everyone else that I work with is straight, and I think carry a fair amount of heterosexism, they are totally accepting of me and my family. For many of them, I am the only gay person they know, and I think that my being out and open about my life has helped them open their minds” (P5) |
| 2 | Authenticity and connections | - “I don't, you know, come out much to patients but you both looked like you'd had a rough time of it. I'm glad I could help. She stood up and pulled Lisa into a hug. Pop down and say hello when you come for your follow up. It would be lovely to see you. I shook her hand, then changed my mind and hugged her as well.” (P3) - She reported that she feels that it's crucial to her sense of self, makes no effort to hide it, and openly talks about her female partner. (P7) |
| 3 | Empowerment / challenging normative assumptions | - Emma said “...when I talk to students, I Bring That Up Too. Being your whole self in space, it always empowers other people, even if you don't know or expect that.” (P2) - when people were very open about their LGBTQ þ identities they were more able to engage in advocacy, for patients/clients and more generally. (P6) - One described confronting a very senior administrator: “[He] made an anti-gay comment in a public forum, and I stood up and protested what he had said.” (P6) |
| 4 | Therapeutic relationships are stronger | - “I had a couple of clients over the years who were themselves LGBTQ, in particular women. And I feel like we had a stronger rapport when they– I guess, I came out to them, but it would just be in a really natural way, like, I probably talked about my partner, and said ‘she’. And then, I just feel like they were much more themselves with me than they might have been with anybody else …”(P6) |
| 5 | Better connection with clients / colleagues | - When participating physical therapists could bring their diverse characteristics into the workplace, they could connect better with patients and foster supportive and inclusive work-places for others, including students, patients, and colleagues. (P2) - …participants noted that identity disclosure could aid rapport, particularly with LGBTQ þ clients/patients: “There's just sort of that common ground and that instant connection with people. So, you kind of already have this built rapport.”(P6) |
