# Supplementary material for: Disclosure experiences in LGBTQ+ healthcare staff: a systematic review and meta-synthesis
Source: BMJ Open. 2026 Mar 4;16(3):e100412. doi: 10.1136/bmjopen-2025-100412 (PMC12970120; doi:10.1136/bmjopen-2025-100412)
Supplement: Supplementary file 4 [file bmjopen-2025-100412.reviewer_comments.pdf]

PEER REVIEW HISTORY

BMJ Open publishes all reviews undertaken for accepted manuscripts. Reviewers are asked to complete a checklist review form and are provided with free text boxes to elaborate on their assessment. These free text comments are reproduced below.

ARTICLE DETAILS

Title (Provisional)

Disclosure experiences in LGBTQ+ healthcare staff: a systematic review and meta-synthesis

Authors

Prakkash, Reem; Manning, Lucy; Bécares, Laia; Hatch, Stephani Louise; Akande, Isaac; Dorrington, Sarah

---

VERSION 1 - REVIEW

**Reviewer**	**1**
**Name**	**Hall, Dani**
**Affiliation**	**Queen Mary University of London, Blizard Institute**
**Date**	**01-Apr-2025**
**COI**	**None**

A clear and comprehensive SR and MA of an important topic where currently a gap in the published literature exists. The authors have conducted a thorough review, creating code and subcodes that appear comprehensive and appropriate to the aims of the study. The authors have included the potential impact of this study with clear recommendations for healthcare employers.

**Reviewer**	**2**
**Name**	**Qualls, Whitney**
**Affiliation**	**Simon Fraser University, Faculty of Health Sciences**
**Date**	**08-Apr-2025**
**COI**	**None**

This review and meta-synthesis describes and examines qualitative data on an under-examined phenomenon in healthcare. The manuscript is clearly written, the analytic approach is appropriate and the conclusions are well rationalized. There are a few minor clarifications and suggestions that I recommend addressing, all found below.

Page 10, lines 54-56: UN

Unclear if all eligible studies happened to take place in the UN or if the UN was a part of the inclusion criteria. If the latter, justification for only including studies from UN is unclear.

Page 11, lines 3-8: Colleague – colleague disclosure

In analysis, provider – patient disclosure experiences are included (page 16, lines 24-26), why were these experiences excluded from the initial search?

Page 30, line 43: Null

Unclear what is meant by a null level of disclosure

Page 35, lines 32-37: Developing country

The term "developing country" is imprecise, unclear what is meant by this classification in this context. How might a country's economic characteristics impact workplace disclosure experiences? The latter part of the sentence ("countries where LGBTQ+ identities are not legalized") better communicates to the reader the characteristics of the country or region where more research is needed on workplace disclosures among healthcare workers.

Page 33, lines 3-36: UK focus

The "Applied" section of the implications seems to be tightly focused on the UK healthcare context. This might be due to the original scope of the proposed paper being limited to the UK healthcare system? Given that the studies reviewed take place both within and outside of the UK, expanding the scope of these implications would be helpful and appropriate.

Page 32, lines 3-57; page 34-35 lines 32-60, 3-13: Bridging sections

It would be helpful if the future recommendations aligned with the implications outlined in the "Applied" section. The future recommendations focus mainly on avenues for future research, but the policy and practice implications that are present in the "Applied" section could be expanded upon in "Recommendations for Future."

**Reviewer**	**3**
**Name**	**Meads, Catherine**
**Affiliation**	**Anglia Ruskin University - Cambridge Campus**
**Date**	**03-Jun-2025**
**COI**	**None**

Abstract: need to change future tense to past. Also, need to make sure you have all of the information required in it according to the PRISMA checklist, as quite a bit missing currently regarding methods used.

Introduction:

The reference to Hunt, et al Being the gay one. is from 2007 so rather old. you could use the BMA report from 2022 - sexual orientation and gender identity in the medical profession, which has similar findings, and obviously little has changed in 15 years.

PICOS refers to patients/population, intervention, comparator, outcome and study design. Your inclusion criteria did not match this, so maybe you should have used a different initialisation?

Thank you for including the protocol for the systematic review, this was very interesting to see. Did you have any systematic reviewers peer review the search strategy? If so they would have pointed out that Medline and Pubmed are the same database on two different platforms, and that Ovid is a platform not a database. It is rare to use pubmed for systematic reviews as it has background coding on that removes potentially includable studies. Better to use Medline on the OVID or EBSCO platform as this provides much better systematic review usability.

The searches you did are listed in the appendix, and you appear to have done four different searches in the OVID platform, each with a variety of databases and journal banks. You state in the methods that each search was on one part of your stated inclusion criteria.

Ordinarily there would be one search in all of the databases at once and including all aspects of your inclusion criteria where practicable. As the searches you did were complicated but not combined, it is impossible to determine whether you have missed any relevant studies or not.

Also, each of the studies ends up with a large number of hits, for example 18216 in one. What did you do with over 18 thousand citations? Did you comb through them all? Did you just use the last one? in which case, why have the previous three in the manuscript?

Also looking through the search strings, it looks like you were only interested in UK evidence. This is not mentioned in the text. However, you do say about studies included were from USA, Canada, Australia etc, so why limit to UK in the search, yet include non-UK studies?

Doing a quick google search, i found the following papers, which may be includable in your systematic review:

https://www.practicenursing.com/content/research/exploring-health-care-professionals-experiences-of-supporting-lgbtqplus-patients-a-qualitative-study/

https://qualitysafety.bmj.com/content/32/2/109

https://bjgp.org/content/71/713/e941

https://www.sciencedirect.com/science/article/pii/S0883941724000177

Sadly, all this adds up to a fatal flaw for a systematic review. It is very likely that you have missed studies, so it doesn't really matter how good the thematic synthesis was (and it looks very good), if several studies are missing, it will only ever be partial.

PRISMA is not a guideline for the conduct of systematic reviews, its an editor's checklist intended to make sure all the relevant details are reported.

What i suggest you do is redo the inclusion criteria and search properly, planned with an experienced systematic reviewer and/or information specialist, redo the inclusions then see how many additional studies you find. Following this it will be clear if the extra studies can be added to the existing thematic synthesis or not. They may well be able to be added because the face validity of the synthesis looks good, and new studies may well show similar themes.

VERSION 1 - AUTHOR RESPONSE

Reviewer 1

**Reviewer's Comments to Authors**	**Authors' response**
A clear and comprehensive SR and MA of an important topic where currently a gap in the published literature exists. The authors have conducted a thorough review, creating code and subcodes that appear comprehensive and appropriate to the aims of the study. The authors have included the potential impact of this study with clear recommendations for healthcare employers.	Thank you.

Reviewer 2

**Reviewer's Comments to Authors**	**Authors' response**
This review and meta-synthesis describes and examines qualitative data on an under-examined phenomenon in healthcare. The manuscript is clearly written, the analytic approach is appropriate and the conclusions are well rationalized. There are a few minor clarifications and suggestions that I recommend addressing, all found below.	Thank you.
Page 10, lines 54-56: UN<br>Unclear if all eligible studies happened to take place in the UN or if the UN was a part of the inclusion criteria. If the latter, justification for only including studies from UN is unclear.	Thank you. We have added the justification for the inclusion criteria as follows:<br>'This ensured the comparability of legal context and focused the analysis on variations in workplace disclosure experiences within states that recognised LGBT rights, avoiding skewing from

	contexts where disclosure was criminalised.’
Page 11, lines 3-8: Colleague – colleague disclosure<br>In analysis, provider – patient disclosure experiences are included (page 16, lines 24-26), why were these experiences excluded from the initial search?	Thank you for this observation. As we indicated in the paper (page 15, lines 12-13), we coded disclosure experiences with patients when they arose, but the focus of our paper is not on the employee-patient relationship. In this paper we focus on the internal workplace experiences of LGBTQ+ healthcare employees. This is in response to a specific gap in the literature. Consequently employee-patient relationships were not included in our search.
**Reviewer’s Comments to Authors**	**Authors’ response**
Page 30, line 43: Null<br>Unclear what is meant by a null level of disclosure	Thank you. We have now clarified this in the paper as follows:<br>‘…with the null level indicating complete absence or non-disclosure of one’s LGBTQ+ identity’
Page 35, lines 32-37: Developing country<br>The term “developing country” is imprecise, unclear what is meant by this classification in this context. How might a country’s economic characteristics impact workplace disclosure experiences? The latter part of the sentence (“countries where LGBTQ+ identities are not legalized”) better communicates to the reader the characteristics of the country or region where more research is needed on workplace disclosures among healthcare workers.	Thank you for highlighting this. We have omitted the ‘developing country’ classification.
Page 33, lines 3-36: UK focus<br>The “Applied” section of the implications seems to be tightly focused on the UK healthcare context. This might be due to the original scope of the proposed paper being limited to the UK healthcare system? Given that the studies reviewed take place both within and outside of the UK, expanding the scope of these implications would be helpful and appropriate.	Thank you for highlighting this. We have now expanded the geographical scope and referenced studies from outside of the UK as well.

Page 32, lines 3-57; page 34-35 lines 32-60, 3-13: Bridging sections<br>It would be helpful if the future recommendations aligned with the implications outlined in the "Applied" section. The future recommendations focus mainly on avenues for future research, but the policy and practice implications that are present in the "Applied" section could be expanded upon in "Recommendations for Future."	Thank you. We have added the applied recommendations from the implications.

Reviewer 3

**Reviewer's Comments to Authors**	**Authors' response**
Abstract: need to change future tense to past. Also, need to make sure you have all of the information required in it according to the PRISMA checklist, as quite a bit missing currently regarding methods used.	Thank you, we have corrected the tense and have ensured the abstract is according to the PRISMA checklist.
Introduction:<br>The reference to Hunt, et al Being the gay one. is from 2007 so rather old. you could use the BMA report from 2022 - sexual orientation and gender identity in the medical profession, which has similar findings, and obviously little has changed in 15 years.	Thank you, we have made that change and have changed it to the BMA report.
PICOS refers to patients/population, intervention, comparator, outcome and study design. Your inclusion criteria did not match this, so maybe you should have used a different initialisation?	We appreciate the observation regarding the use of the PICOS framework and addressing this concern: while PICOS (Population, Intervention, Comparator, Outcome, Study Design) is typically applied to quantitative studies, our review followed the PICoS model (Population, Phenomenon of Interest, Context, and Setting), commonly recommended for qualitative evidence synthesis. This allowed us to more appropriately define our inclusion criteria, focusing on LGBTQ+ healthcare professionals and their experiences of disclosure in workplace settings.<br>References:<br>• https://systematicreviewsjournal.biomedcentral.com/articles/10.1186/s13643-016-0249-x/tables/4

**Reviewer's Comments to Authors**	**Authors' response**
	• Stern, C., Jordan, Z., & McArthur, A. (2014). Developing the review question and inclusion criteria. *AJ The American Journal of Nursing, 114*(4), 53-56.<br>• Hosseini, M. S., Jahanshahlou, F., Akbarzadeh, M. A., Zarei, M., & Vaez-Gharamaleki, Y. (2024). Formulating research questions for evidence-based studies. *Journal of Medicine, Surgery, and Public Health, 2*, 100046.
Thank you for including the protocol for the systematic review, this was very interesting to see. Did you have any systematic reviewers peer review the search strategy? If so they would have pointed out that Medline and Pubmed are the same database on two different platforms, and that Ovid is a platform not a database. It is rare to use pubmed for systematic reviews as it has background coding on that removes potentially includable studies. Better to use Medline on the OVID or EBSCO platform as this provides much better systematic review usability.	Thank you for your comments and the opportunity to clarify our use of the different search engines and platforms.<br><br>Thank you for highlighting the overlap between Medline and Pubmed. To clarify, we did not use PubMed following our initial protocol development. We conducted a brief search in PubMed and as expected did not identify any additional relevant studies beyond those retrieved through MEDLINE via the Ovid platform. While OVID is a platform, we accessed multiple databases through it (e.g., PsycINFO, Medline), as specified in the manuscript.<br><br>Our search strategy was developed with research support from the Masters in Organisational Psychiatry and supervised by an experienced systematic reviewer. Our search strategy was then reviewed by two senior co-authors who also have extensive experience of undertaking and supervising systematic reviews. The search strategy and paper were then reviewed as part of the Masters and approved and awarded a distinction.<br><br>In response to your comments, we asked King's College London Research Support Team to review and comment on the search before re-submitting the updated manuscript. The KCL team reviewed and approved the search.<br><br>Please let us know if you need further clarification.

Reviewer's Comments to Authors	Authors' response
The searches you did are listed in the appendix, and you appear to have done four different searches in the OVID platform, each with a variety of databases and journal banks. You state in the methods that each search was on one part of your stated inclusion criteria. Ordinarily there would be one search in all of the databases at once and including all aspects of your inclusion criteria where practicable. As the searches you did were complicated but not combined, it is impossible to determine whether you have missed any relevant studies or not. Also, each of the studies ends up with a large number of hits, for example 18216 in one. What did you do with over 18 thousand citations? Did you comb through them all? Did you just use the last one? in which case, why have the previous three in the manuscript?	Thank you for giving us an opportunity to make the paper clearer. As described in detail under Selection and Data Extraction Process, we followed the approach outlined by Ranganathan and Aggarwal (2020). Our initial step was to screen the abstracts of papers retrieved from the database searches. Given the large number of studies identified in the final search (n = 4,087 through Ovid), we introduced an additional preliminary step of screening papers based on their titles before moving to abstract review.<br><br>While four searches were conducted, the first three served as scoping exercises that informed the development of the fourth and final search string. Only the results from this final search were deduplicated (n = 4,087) and used for screening, ensuring a comprehensive and consistent process.<br><br>The full search strategies are provided in the Supplementary Material 1of our original submission. We first applied a title-screening step to exclude clearly irrelevant papers, followed by full abstract screening for the remaining records.<br><br>In conclusion, we agree with your suggestion to remove the first three scoping searches to prevent confusion for future readers. These were part of the original Masters thesis that required extra detail to be included are not required in this context.
Also looking through the search strings, it looks like you were only interested in UK evidence. This is not mentioned in the text. However, you do say about studies included were from USA, Canada, Australia etc, so why limit to UK in the search, yet include non-UK studies?	That is correct, we did include international studies. To clarify - we did not limit to UK in the search.<br><br>We included some UK-specific terms (e.g., NHS) but these terms were not used as filters or limitations to restrict results solely to UK studies. We therefore included globally relevant terms, and eligible studies came from countries that endorsed the 2011 UN LGBT Rights Declaration (e.g., USA, Canada, Australia). The inclusion of non-UK studies is thus consistent with our stated eligibility criteria.

Reviewer's Comments to Authors	Authors' response
Doing a quick google search, i found the following papers, which may be includable in your systematic review:<br>• https://www.practicenursing.com/content/research/exploring-health-care-professionals-experiences-of-supporting-lgbtqplus-patients-a-qualitative-study/<br>• https://qualitysafety.bmj.com/content/32/2/109<br>• https://bjgp.org/content/71/713/e941<br>• https://www.sciencedirect.com/science/article/pii/S0883941724000177	Thank you and we appreciate the suggested articles however, of the four papers mentioned:<br>• The first three focus on patient care or perspectives<br>• The fourth falls outside our 2011-2023 publication window.<br><br>These were therefore excluded in line with our predefined criteria.

VERSION 2 - REVIEW

**Reviewer**	**2**
**Name**	**Qualls, Whitney**
**Affiliation**	**Simon Fraser University, Faculty of Health Sciences**
**Date**	**06-Nov-2025**
**COI**	

Comments satisfactorily addressed

**Reviewer**	**3**
**Name**	**Meads, Catherine**
**Affiliation**	**Anglia Ruskin University - Cambridge Campus**
**Date**	**28-Oct-2025**
**COI**	

Many thanks for submitting this manuscript on workplace disclosure of LGBTQ+ identity by healthcare employees.

In the background you describe how "roughly 38% of sexual and gender minority healthcare staff conceal their identity in the workplace" - this must depend on the country you work in. for example in Nigeria I bet the proportion is very close to 100% concealment. Are there any

references around the range of concealment in different countries? Also i think you need to distinguish between disclosure to other staff and disclosure to patients. Can you cite any evidence on this? For example this is some in the BMA/GLADD report -Sexual orientation and gender identity in the medical profession 2022.

Why did you only include studies published in English? Most of the countries that are United Nations members and have endorsed LGBT rights do not use English. Given the low number of included studies, it would be useful to know if any relevant studies have been missed.

The searches used the following databases (platforms): PsycInfo (OVID); Journals@Ovid Full Text; Social Policy and Practice (OVID); PsycArticles (OVID). I don't understand why Medline and Embase were not searched, as these may well have yielded more includable studies. Cinahl would have been another one to search, given that you were looking for health worker studies.

In your responses to the peer review comments first time you state: 'While

OVID is a platform, we accessed multiple databases through it (e.g., PsycINFO, Medline), as specified in the manuscript.'

But you don't list Medline in the manuscript. Did you search it or not?

4087 citations before duplicate removal was not a high number when doing this kind of a systematic review.

I'm not sure of the usefulness of table 3.

In your searches reproduced in the appendix you have the numbers incurrect at the end:

31 14 and 29 and 30 5683

32 limit 36 to english 5682

33 limit 37 to transgender 5640

34 limit 38 to yr="2011 -Current" 4087

If you only have 34 lines, how can you limit line 38?

How did each paper contribute to the themes you generated. ideally i would have liked to see a CerQUAL table demonstrating this.

Given this is the second peer review, i'm not convinced that all of the original round of peer review has actually been addressed regarding the methods.

VERSION 2 - AUTHOR RESPONSE

Reviewer 3

Reviewer's Comments to Authors	Author's Response
In the background you describe how "roughly 38% of sexual and gender minority healthcare staff conceal their identity in the workplace" - this must depend on the country you work in. for example in Nigeria I bet the proportion is very close to 100% concealment. Are there any references around the range of concealment in different countries?	Thank you for this helpful comment and we agree about geographical location shaping workplace disclosure experiences. We have now specified that the cited study by Eliason et al. (2017) involved respondents from U.S. professional networks, thereby clarifying the context of the reported estimate.
Also i think you need to distinguish between disclosure to other staff and disclosure to patients. Can you cite any evidence on this? For example this is some in the BMA/GLADD report -Sexual orientation and gender identity in the medical profession 2022.	Thank you for highlighting this point. Our review was driven by PPI input to explore workplace experiences among colleagues, and the analysis was deliberately limited to disclosure to professional peers rather than to patients, given the different contexts and implications of these types of disclosure.
Why did you only include studies published in English? Most of the countries that are United Nations members and have endorsed LGBT rights do not use English. Given the low number of included studies, it would be useful to know if any relevant studies have been missed.	Thank you for this valuable comment. As the review involved detailed qualitative extraction and thematic synthesis, we limited inclusion to English-language studies to ensure methodological rigour and accuracy in coding and interpretation. We acknowledge that this approach may have resulted in the exclusion of some relevant studies and have now noted this as a limitation in the revised manuscript.
The searches used the following databases (platforms): PsycInfo (OVID); Journals@Ovid Full Text; Social Policy and Practice (OVID); PsycArticles (OVID). I don't understand why Medline and Embase were not searched, as these may well have yielded more includable studies. Cinahl would have been another one to search, given that you were looking for health worker studies.	Thank you for this observation. We understand your concern and so have rerun a wider search which has not found any missed papers.<br><br>The final search strategy prioritised PsycINFO, PsycARTICLES, Journals@Ovid Full Text, and Social Policy and Practice, which were considered most closely aligned with the focus of the review. To minimise the risk of missing eligible studies beyond these databases, comprehensive supplementary search methods were employed, including forward and backward citation tracking and parallel searches in Google Scholar and Scopus. Together, these approaches were intended to

Reviewer’s Comments to Authors	Author’s Response
	ensure comprehensive coverage in the final database searches.
In your responses to the peer review comments first time you state: 'While OVID is a platform, we accessed multiple databases through it (e.g., PsycINFO, Medline), as specified in the manuscript.' But you don't list Medline in the manuscript. Did you search it or not?	Thank you for highlighting this point. The initial scoping searches (searches 1–3) did include MEDLINE via Ovid. In practice, the MEDLINE and PubMed searches we conducted separately did not yield results particularly relevant to our focus on psychological and workforce studies, which is why the final search was adapted to concentrate on the other databases.<br><br>In an earlier version of the manuscript and supplementary materials, Medline was mentioned when all preliminary scoping search strings were provided for transparency. Following the helpful comments received during the first round of peer review, the manuscript was revised to report only the final search strategy and databases used in the definitive search execution. As Medline was not included in the final search, it is no longer listed in the revised manuscript. The databases currently reported therefore accurately reflect those used in the final search.
4087 citations before duplicate removal was not a high number when doing this kind of a systematic review.	Thank you for this observation. While the initial 4087 citations may seem modest, we conducted an expanded and comprehensive search, as also described in our Methods section, to ensure full coverage. This included systematic hand searching, forward and backward citation tracking of relevant papers, and searching additional databases such as Google Scholar and Scopus. Through this multi-faceted approach, we are confident that all relevant studies were identified and that no eligible papers were missed.

Reviewer's Comments to Authors	Author's Response
I'm not sure of the usefulness of table 3.	Thank you for this suggestion. Table 3 was included as a reference for all included studies and to link each study to its corresponding code in the thematic synthesis, making it easier for readers to navigate the findings.
In your searches reproduced in the appendix you have the numbers incurrect at the end:<br>31 14 and 29 and 30 5683<br>32 limit 36 to english 5682<br>33 limit 37 to transgender 5640<br>34 limit 38 to yr="2011 -Current" 4087<br>If you only have 34 lines, how can you limit line 38?	Thank you very much for this helpful comment and for drawing our attention to the discrepancy in the search documentation.<br><br>When revisiting our original search files, we noticed that the number of lines shown in the exported Appendix did not fully match the numbering displayed when the search was rerun in Ovid. This appears to result from how Ovid manages search histories, where rerunning searches or applying limits can renumber or consolidate sets rather than retain all original line numbers, as documented by Ovid (https://tools.ovid.com/ovidtools/medline/). We have now corrected the numbers for clarity.
How did each paper contribute to the themes you generated. ideally i would have liked to see a CerQUAL table demonstrating this.	Thank you for this helpful comment. Each paper contributed to the themes through detailed extraction and coding of relevant findings by two independent coders, which were then synthesised across studies to generate the overarching themes. While a CerQUAL table could further illustrate the contribution of each study, the thematic synthesis was designed to capture patterns across the body of evidence rather than individual study mapping.

VERSION 3 - REVIEW

Reviewer **3**

Name **Meads, Catherine**

Affiliation **Anglia Ruskin University - Cambridge Campus**

**Date**	**21-Jan-2026**
**COI**	

see above
